# Exogenous growth factors bFGF, EGF and HGF do not influence viability and phenotype of ^V600E^BRAF melanoma cells and their response to vemurafenib and trametinib *in vitro*

**DOI:** 10.1371/journal.pone.0183498

**Published:** 2017-08-22

**Authors:** Izabela Zalesna, Marta Osrodek, Mariusz L. Hartman, Michal Rozanski, Malgorzata Sztiller-Sikorska, Karolina Niewinna, Dariusz Nejc, Malgorzata Czyz

**Affiliations:** 1 Department of Molecular Biology of Cancer, Medical University of Lodz, Lodz, Poland; 2 Department of Surgical Oncology, Medical University of Lodz, Lodz, Poland; University of Queensland Diamantina Institute, AUSTRALIA

## Abstract

It has been shown that the response of ^V600E^BRAF melanoma cells to targeted therapeutics is affected by growth factors. We have investigated the influence of three different growth factors, bFGF, EGF and HGF used either alone or in combination, on the response of ^V600E^BRAF melanoma cell populations established from surgical specimens to vemurafenib and trametinib, targeting ^V600E^BRAF and MEK1/2, respectively.

We report that proliferation and phenotype of ^V600E^BRAF melanoma cell populations were not detectably influenced by exogenous growth factors. Neither cell distribution in cell cycle and *CCND1* expression nor activity of signaling pathways crucial for melanoma development and maintenance, including the RAF/MEK/ERK pathway, WNT/β-catenin pathway and NF-κB signaling, were affected by the presence of different growth factors. We furthermore show that *v*emurafenib and trametinib abrogated the activity of ERK1/2, arrested cells in G_0_/G_1_ cell cycle phase, triggered apoptosis, induced changes in the expression of *CXCL8*, *CCND1* and *CTGF* and the frequency of Ki-67^high^ and CD271^high^ cells. These effects were, however, similar in the presence of different growth factors. Interestingly, comparable results were also obtained for melanoma cells grown without exogenous growth factors bFGF, EGF and HGF for a period as long as 4 months prior the drug treatment.

We conclude that the composition or lack of exogenous growth factors bFGF, EGF and HGF do not markedly influence viability and phenotype of ^V600E^BRAF melanoma cells and their response to vemurafenib and trametinib *in vitro*. Our results question the necessity of these growth factors in the medium that is used for culturing ^V600E^BRAF melanoma cells.

## Introduction

The present study on the influence of growth factors on melanoma cell phenotype and response to vemurafenib (Zelboraf) and trametinib (Mekinist) was justified by recently published reports. It has been demonstrated that untreated ^V600E^BRAF melanoma cells exhibit high levels of feedback inhibition in the RAF/MEK/ERK signaling pathway that diminishes the ability of receptor tyrosine kinase signaling to activate ERK [1]. This feedback inhibition becomes deregulated in vemurafenib-treated cells, which increases cell responsiveness to growth factors, such as bFGF, HGF and EGF [1]. Moreover, it has been shown that ligands, especially HGF can limit the response to a vemurafenib analogue [2]. Interestingly, vemurafenib treatment increased the fibroblast-mediated release of HGF [3], and HGF secretion by stroma cells evoked resistance to RAF inhibitors [2,4,5] or as suggested by Filitis et al., just induced HGF-mediated mitogenesis [6].

It is already recognized that cancer cells derived from tumor specimens and shortly cultured *in vitro* still preserve individual tumor properties. However, this *in vitro* approach, which is considered as having a great potential to exclude ineffective patient treatment regimens, suffers from lack of sufficient amount of cells to cover all necessary assessments to yield conclusive and consistent results on individualized drug treatment that can be applied in the clinics. Working on preserving individual tumor characteristics in an *in vitro* approach, we have already demonstrated that serum-containing medium largely affects the original melanoma cell phenotype. On the contrary, culturing patient-derived melanoma cell populations in stem cell medium (SCM) containing basic fibroblast growth factor (bFGF) and epithelial growth factor (EGF) better preserves the original tumor characteristics [7–10]. Several genes including *MITF-M* and MITF-dependent genes were expressed in SCM and original tumors at similar levels [8]. We have already used melanoma cell populations established from surgical specimens and grown in SCM to select natural compounds most potent in affecting the self-renewing capacity of melanoma cells [11] and to evaluate drug penetration capacity into spheroid structure [12]. One of the natural compounds, parthenolide has been shown to induce extracellular signal-regulated kinase ½ (ERK1/2) activity, MITF-M downregulation and senescence in MITF-M^high^ melanoma cell populations [13]. Pentoxyfilline, another natural drug that is approved by FDA in the treatment of peripheral arterial disease, has shown a capacity to downregulate the WNT/β-catenin pathway in β-catenin^high^ melanoma cells [14], which might be further explored in a subset of melanoma patients not responding to immunotherapy [15] or targeted therapy [16] due to a high activity of this pathway. We think that all these results might be overlooked when more uniform melanoma cell populations, such as monolayers grown in the medium containing serum, are used. In the present report, we further explored the influence of growth conditions on melanoma cells and cell response to treatment with vemurafenib (Zelboraf) targeting ^V600E^BRAF and trametinib (Mekinist) targeting mitogen-activated kinase/ERK kinase 1/2 (MEK1/2). Three different growth factors, bFGF, EGF and hepatocyte growth factor (HGF) were used, either alone or in combination to reveal whether growth factors or their particular combination can influence the melanoma cell response to targeted therapy. We used drug-naïve melanoma populations derived from surgical specimens. They were recently well characterized on the molecular and cellular level [17]. All of them harbor a mutation in *BRAF* resulting in an active ^V600E^BRAF kinase. As expected, these cells responded to vemurafenib and trametinib with reduced proliferation, which was accompanied by attenuated activity of ERK1/2 and nuclear factor κB (NF-κB). Expression levels of interleukin 8 (IL-8), which exhibits significant overexpression in melanoma [18] substantially reduced during BRAF inhibitor treatment [19] and connective tissue growth factor (CTGF), recently considered as a therapeutic target for metastatic melanoma [20], were also significantly reduced by vemurafenib and trametinib in naïve melanoma populations derived from surgical specimens [17]. Drug-decreased percentage of Ki67^high^ cells indicates reduced proliferation rate, whereas increased percentages of nerve growth factor receptor (CD271)^high^ cells suggest selection of melanoma stem-like cells by vemurafenib and trametinib [17].

In the second part of our study, knowing that activated kinase ^V600E^BRAF drives the growth factor-independent activation of the ERK1/2 pathway, we also asked the question whether exogenous growth factors are necessary at all for melanoma cell growth, and how lack of them in the culture medium might influence melanoma cell response to targeted therapy.

## Materials and methods

### Drugs

Vemurafenib and trametinib were from Selleck Chemicals LLC (Houston, TX, USA).

### Tumor tissues and cell culture

Melanoma cell populations derived from drug-naïve patient tumors were investigated. The study was approved by Ethical Commission of Medical University of Lodz. Informed consent was obtained from all patients. Cells were named DMBC after Department of Molecular Biology of Cancer with consecutive numbers. Melanoma cells were isolated and maintained as described previously [9]. Shortly, after several washes, tumor fragments were minced with scissors and incubated in HBSS (SigmaAldrich, St Louis, MO, USA) supplemented with 3 mM calcium chloride and 1 mg/ml collagenase IV for 2–3 h at 37°C. DNase I (10 μg/ml) was added and cells were filtered through a 70 μm pore size filter. Cells were cultured in complete medium (RPMI-1640 with 10% FBS) for 1 day to remove dead and nonadherent cells. They were transferred to serum-free stem cell medium (SCM), consisting of DMEM/F12 low osmolality medium (Lonza, Basel, Switzerland), B-27 supplement (Gibco, Paisley, UK), insulin (10 μg/ml), heparin (1 ng/ml), 10 ng/ml bFGF, 20 ng/ml EGF (BD Biosciences, San Jose, CA, USA) and antibiotics (100 IU/ml penicillin, 100 μg/ml streptomycin).

A study design.

In the first part of the study focusing on the influence of different growth factors, melanoma cells were grown in the medium consisting of DMEM/F12, B-27, insulin, heparin and antibiotics supplemented with 10 ng/ml bFGF, 20 ng/ml EGF and 40 ng/ml HGF (Gibco, Frederick, MD, USA), used either alone or in combination for 10 days prior a drug treatment. In the second part of the study, melanoma cells were grown in the medium containing the same supplements as SCM except for growth factors for up to 4 months prior a drug treatment. For experiments, melanoma cells were left to grow overnight in appropriate medium before being treated with 10 μM vemurafenib (PLX) or 50 nM trametinib (TRA). In both parts of the study, melanoma cells grown in SCM served as a control. Melanoma cells were collected after 24 h of incubation with drugs for RNA isolation, and after 44 h for protein lysates, Annexin V/PI staining, cell cycle and immunophenotype analysis. Culture medium was frozen for the IL-8 and HGF secretion analysis.

### Microscopy

Changes in cell morphology were registered with a digital Olympus camera (C-5050) attached to Olympus microscope (CKX41, Olympus, London, UK).

### Time-lapse fluorescence microscopy

For cell proliferation and apoptosis, a time-lapse fluorescence microscope system (IncuCyte, Essen Bioscience) was used. The data were analyzed using IncuCyte Zoom original software. Proliferation was assessed as changes in the area occupied by melanoma cells in control and drug-treated cultures (% of confluence) over time. For apoptosis assay, melanoma cells were seeded in 96-well plates at 8 x 10^3^ cells/well with IncuCyte^™^ Caspase-3/7 Apoptosis Assay Reagent at 4 μM and exposed to 10 μM vemurafenib, 50 nM trametinib or vehicle for 3 days. Activation of caspase-3/7 was monitored every 3 h using live cell imaging and quantified using the IncuCyte^®^ ZOOM basic analyser. It was expressed as % of confluence of apoptotic cells divided by % of confluence of all cells.

### Flow cytometry

Melanoma cells were left to grow overnight before being treated with drugs for 44 h. In immunophenotype analysis, isotype controls were included in each experiment. To exclude dead cells from the analysis, LIVE/DEAD^®^ Fixable Violet Dead Cell Stain Kit (Life Technologies, Eugene, OR, USA) was used. Antibodies against CD271 (PE-conjugated) and Ki-67 (Alexa Fluor 647-conjugated) were from BD Pharmingen (San Jose, CA, USA). For intracellular staining, cells were fixed with 4% paraformaldehyde and permeabilized with 0.1% Triton X-100 in PBS for 20 min. Flow cytometric data were acquired with FACSVerse (BD Biosciences, San Jose, CA, USA), and analyzed using BD FACSuite. For cell cycle analysis, PI/RNase Staining Buffer (BD Pharmingen) was used. ModFit LT 3.0 software was used to calculate the percentages of cells in each cell cycle phase.

Detection of apoptosis was carried out by dual staining with Annexin V-FITC and PI (Roche Diagnostics, Manheim, Germany). Melanoma cells were seeded into 12-well plates and treated with drugs for 44 h. After treatment, cells were collected, centrifuged at 400 x g for 5 min and stained with Annexin V-FITC and PI for 15 min at room temperature in the dark. 30,000 events were analyzed for each sample by flow cytometer FACSVerse (Becton Dickinson), and results were processed by using FACSuite software (Becton Dickinson).

### RNA isolation, cDNA synthesis, and quantitative RT-PCR (qRT-PCR)

RNA was isolated and purified using Total RNA Isolation kit with mini column system (A&A Biotechnology, Gdynia, Poland). Concentration and purity of RNA samples were determined using a NanoQuant plate reader (Infinite M200Pro, Tecan). Total RNA (1 μg) was transcribed into cDNA using 300 ng of random primers and SuperScript II Reverse Transcriptase (Invitrogen Life Technologies, Carlsbad, CA, USA). The evaluation of mRNA expression of selected genes was performed by quantitative RT-PCR using the Rotor-Gene 3000 Real-Time DNA analysis system (Corbett Research, Morklake, Australia). Amplification was performed using KAPA SYBR FAST qPCR Kit Universal 2X qPCR Master Mix (Kapa Biosystems, Cape Town, South Africa), 200 nM of each primer and 25 ng cDNA template per reaction. Sequences of primers used in qRT-PCR were shown elsewhere [8,21]. To calculate the relative expression of target genes *versus* a reference gene *RPS17*, a mathematical model including an efficiency correction was used.

### Western blots

Melanoma cells were lysed in RIPA buffer containing 50 mmol/l Tris-HCl pH 8.0, 150 mmol/l NaCl, 1% Triton X-100, 0.5% sodium deoxycholate, 0.1% SDS supplemented with freshly added protease and phosphatase inhibitors (Sigma-Aldrich). Protein concentration was determined by Bradford assay. Cell lysates were diluted in 2x Laemmli buffer and protein samples (15 μg each) were loaded on 7% SDS-polyacrylamide gel. The proteins were transferred onto an Immobilon-P PVDF membrane (Millipore, Billerica, MA, USA). The membrane was incubated in a blocking solution: 5% nonfat milk in PBS-Tween 0.05% or 5% phosphoBLOCKER (Cell Biolabs, San Diego, CA, USA) in PBS-Tween 0.05%. Primary antibodies detecting PARP and total β-catenin were from Santa Cruz Biotechnology, active β-catenin (dephosphorylated on Ser^37^ and Thr^41^) from Millipore (Temecula, CA, USA), p-p65 (Ser^536^), p65, p-ERK1/2 (Thr^202^/Tyr^204^), ERK1/2 from Cell Signaling Technology (Danvers, MA, USA). Immunodetection of β-actin (Sigma-Aldrich) was used as a loading control.

### Enzyme-linked immunosorbent assay (ELISA)

ELISA kit Quantikine High Sensitivity Human CXCL8/IL-8 and ELISA kit Quantikine Human HGF (R&D Systems, Minneapolis, MN, USA) were used to determine IL-8 and HGF secretion to the culture medium, respectively. Assays were performed according to the manufacturers’ instructions. The optical density of each well was determined using a microplate reader Infinite M200Pro (Tecan). Concentrations of IL-8 and HGF in the medium samples were obtained by using a four parameter logistic (4-PL) curve fit.

### Statistical analysis

To calculate statistical significance of differences, Statistica v.12 software was used. Normality of a sample distribution was assessed with Shapiro-Wilk test. Equality of variances between two or more samples was checked with Levene's test. To compare two samples with normal distribution and equal variances Student's T test was used, in case of unequal variances—Welch F test. If sample was displaying non-normal distribution or when n = 2, Mann-Whitney U test was used. To compare 3 or more samples with normal distribution and equal variances, ANOVA was used. In case of lack of normality, inequality of variances or n = 2, Kruskal-Wallis H test was used. Statistically significant results were considered at *P* < 0.05.

## Results

### Composition of exogenous growth factors (bFGF, HGF and EGF) does not substantially influence proliferation of ^V600E^BRAF melanoma cells

**Fig 1:**
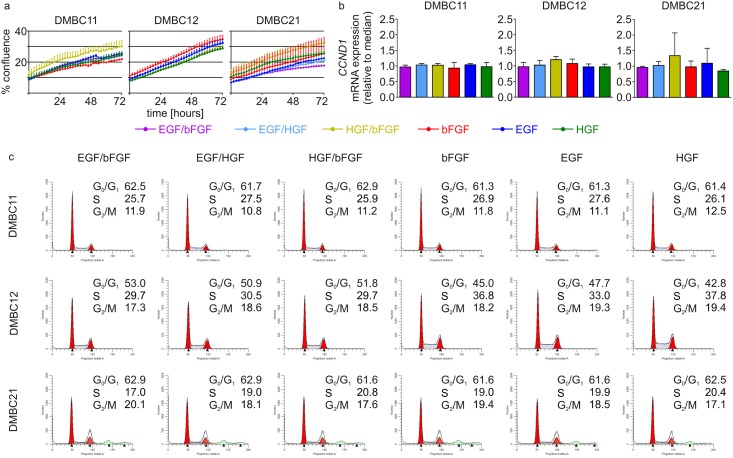
Composition of exogenous growth factors (bFGF, HGF and EGF) does not markedly influence ^V600E^BRAF melanoma cell proliferation, expression of *CCND1* and cell distribution in the cell cycle. **a.** Cell proliferation was monitored by analyzing the occupied area (% confluence) of cell images over time using IncuCyte. **b.** Basal expression level of *CNND1* was assessed by qRT-PCR and normalized to the expression of a reference gene *RPS17*. Gene expression level in each melanoma cell population was expressed relatively to the median values. Bars represent the mean value ± SD. No statistically significant differences were found (S1 Table). **c.** Cell cycle profiles were determined by flow cytometry. Representative histograms and their quantification from a representative experiment are shown. ModFit LT 3.0 software was used to calculate the percentages of viable cells in cell cycle phases. DMBC, patient-derived melanoma cell populations obtained in the Department of Molecular Biology of Cancer.

Growth factors, HGF, bFGF and EGF were used in the culture medium for 10 days, either alone or in combination. Cell proliferation was investigated in 3 melanoma cell lines established from surgery specimens, DMBC11, DMBC12 and DMBC21 using IncuCyte (Fig 1a). This imagining system allows to register changes in cell culture in real time. No substantial differences were observed between cell proliferation curves obtained for cell cultures grown in the medium containing bFGF, EGF, HGF, used either alone or in combination (Fig 1a). Expression of *CCND1*, a target gene of the ERK1/2 pathway encoding cyclin D1, a main protein required for progression through the G_1_ phase of the cell cycle, was assessed at the level of transcript (Fig 1b). No differences in *CCND1* expression were observed in all three cell populations grown in different conditions (Fig 1b). Cell distribution in cell cycle phases was also very similar when melanoma cells were grown in the media containing different ligands (Fig 1c).

### Composition of exogenous growth factors (bFGF, HGF and EGF) does not markedly influence the response of ^V600E^BRAF melanoma cells to vemurafenib and trametinib

**Fig 2:**
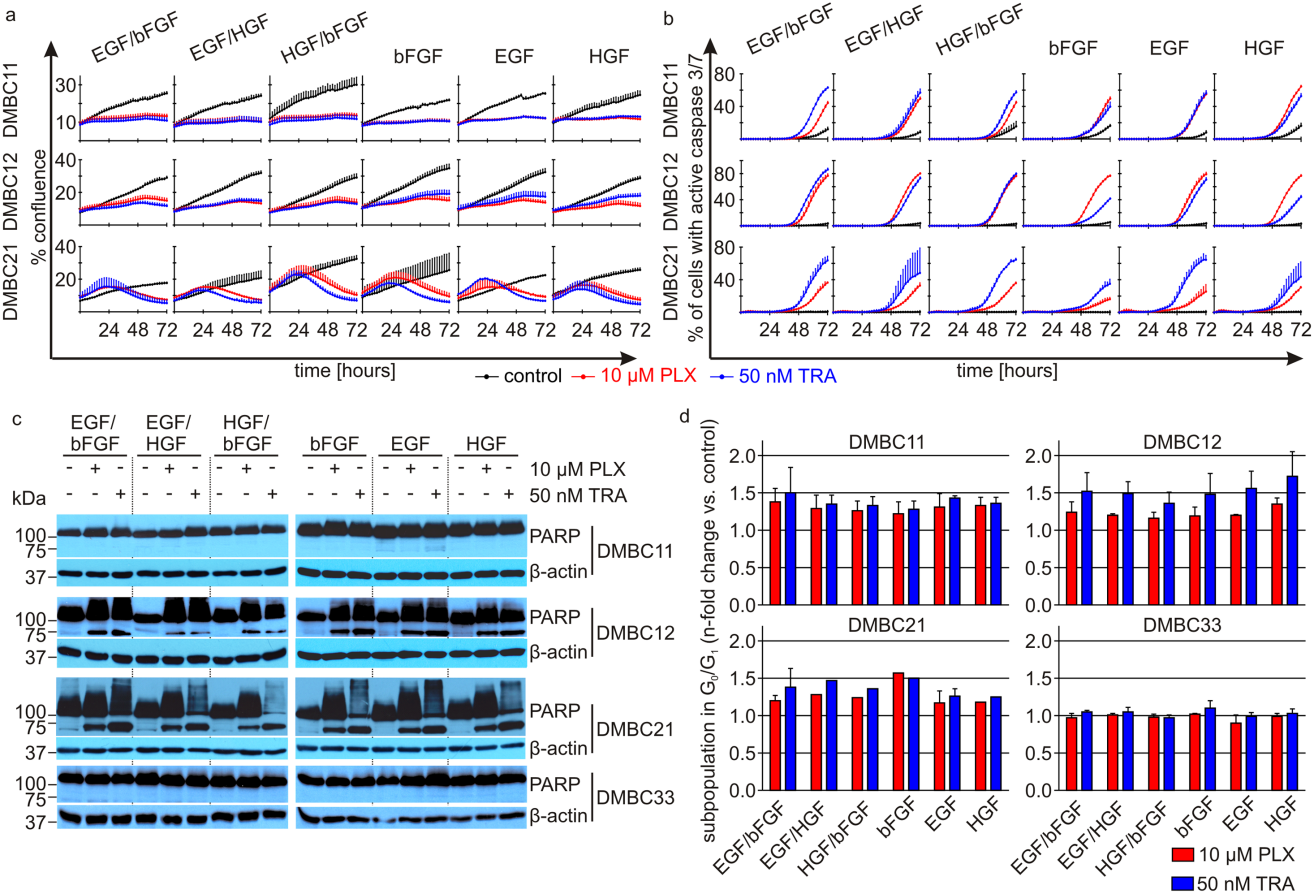
Composition of exogenous growth factors (bFGF, HGF and EGF) does not substantially affect vemurafenib (PLX)- and trametinib (TRA)-induced changes in melanoma cell proliferation, the induction of apoptosis and cell cycle arrest in G_0_/G_1_. **a.** Proliferation time-courses. ^V600E^BRAF melanoma cell proliferation was monitored by analyzing the occupied area (% confluence) of cell images over time using IncuCyte. Effects of 10 μM PLX and 50 nM TRA were observed in all tested populations, and reached similar levels regardless of growth conditions. **b. c.** Apoptosis induced by PLX and TRA in melanoma cells grown in the presence of different growth factors. **b.** Percent of apoptotic cells with high caspase 3/7 activity was assessed in time-lapse imaging system IncuCyte over the course of 72 h. **c.** Apoptosis shown as PARP cleavage after 44 h of incubation with drugs. β-actin was used as a loading control. **d.** Distribution of melanoma cells in cell cycle phases was determined by flow cytometry. ModFit LT 3.0 software was used to calculate the percentages of viable cells in cell-cycle phases. Drug-induced cell accumulation in G_0_/G_1_ phase was shown relatively to the respective control. Bars represent the mean values ± SD. The histograms for DMBC12 and DMBC33 cells are shown in S1 Fig.

Proliferation of DMBC11, DMBC12 and DMBC21 cell populations was affected to a similar extent by 10 μM vemurafenib and 50 nM trametinib irrespective of the composition of growth factors used in culture media during 72 h of incubation (Fig 2a). The magnitude of induction of apoptotic cell death in different growth conditions measured as a real-time increase of caspase 3/7 activity (Fig 2b) and PARP cleavage after 44 h of treatment (Fig 2c) varied across the experiments and the melanoma cell populations examined but no substantial differences were observed for different compositions of growth factors. Apoptosis was not induced at lower concentrations of drugs as shown for DMBC28 cell population (S1 Fig). We assessed PARP cleavage also in DMBC33 cell population, which did not respond to vemurafenib and trametinib in the medium containing both bFGF and EGF [17], and the same lack of effect was observed in the presence of all tested growth factors used either alone or in combination (Fig 2c). As it has been demonstrated that vemurafenib and trametinib induce cell cycle arrest in G_0_/G_1_ phase, a drug-triggered n-fold increase in the percentage of cells in G_0_/G_1_ phase was calculated for each growth condition (Fig 2d). Again, there was no substantial differences between populations grown in the presence of different growth factors. Of note, in slow cycling DMBC33 population, in which a high percentage of untreated (control) cells were already arrested in G_0_/G_1_ phase, no further accumulation in this cell cycle phase was observed (Fig 2d and S2 Fig).

**Fig 3:**
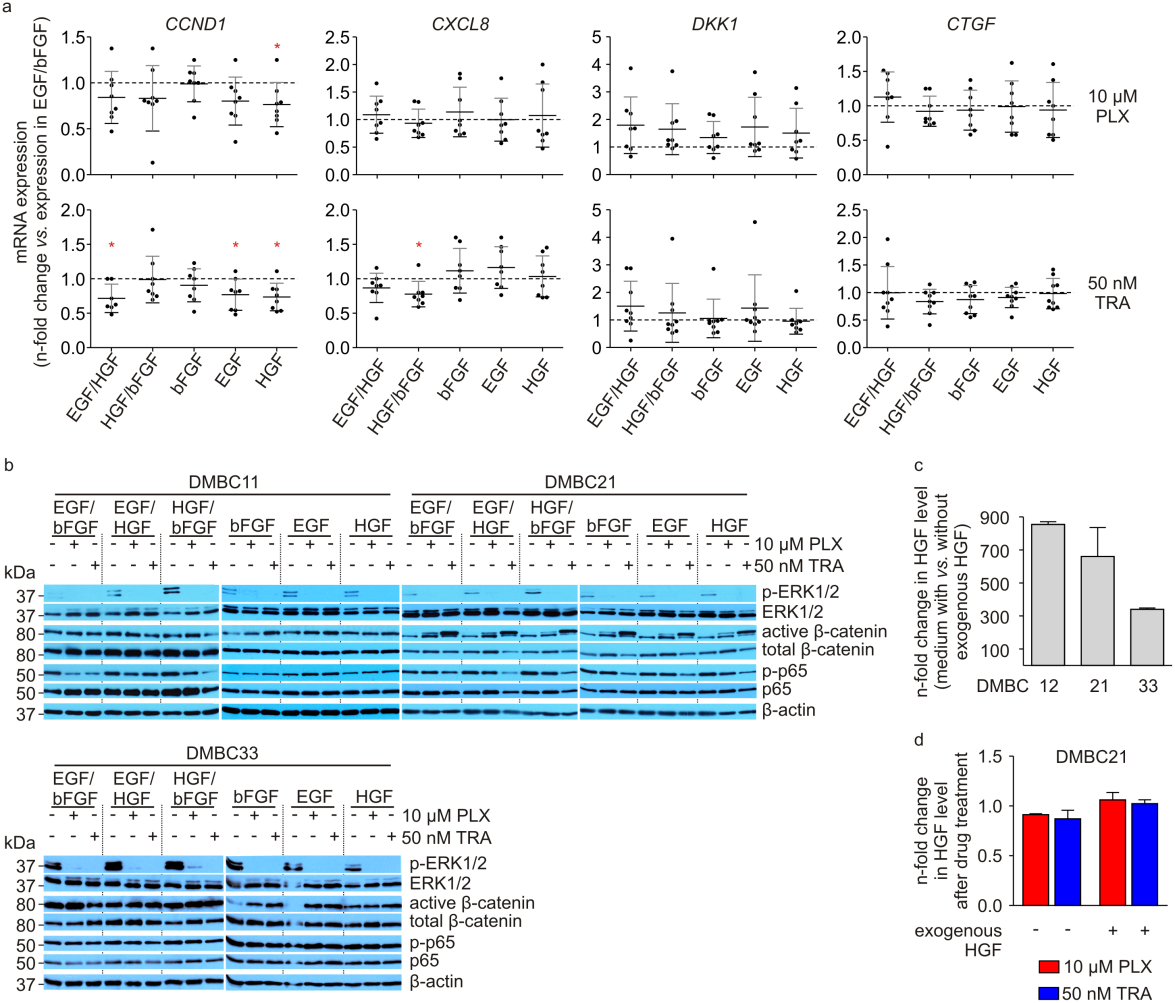
Effects of vemurafenib (PLX) and trametinib (TRA) on gene expression and protein activities in melanoma cell populations grown in the presence of different growth factors or their combinations. **a.** qRT-PCR validation of changes in the expression *of CCND1*, *CXCL8*, *DKK1* and C*TGF* in melanoma cells after 24 h of incubation with 10 μM PLX and 50 nM TRA. The expression is normalized to the expression of a reference gene *RPS17*. A fold change in the drug-induced alteration of gene expression in melanoma cells grown in media containing different growth factors relatively to the alteration of expression in cells grown in SCM containing bFGF and EGF. Points indicate individual values obtained for respective growth conditions, and horizontal lines represent means for pooled values. Differences are considered significant at *P < 0.05. **b.** Melanoma cells were treated with 10 μM PLX and 50 nM TRA for 44 h and the levels of phosphorylated ERK1/2 (p-ERK1/2), total ERK1/2, active β-catenin, total β-catenin, phosphorylated p65 (p-p65) and p65 were assessed by immunoblotting. Equal loading was confirmed by β-actin. Representative results are shown. **c**. The level of HGF was compared between the medium with exogenous *vs*. medium without exogenous HGF after 24 h of culturing of untreated cells. Bar graphs represent the mean values ± SD. **d**. Melanoma cells were treated with 10 μM PLX and 50 nM TRA for 24 h, the level of HGF was assessed in the culture media and expressed relatively to the level in the untreated culture, in which the level of HGF was set as 1. Bar graphs represent the mean values ± SD.

On the transcript level, expression of *CTGF* and *CXCL8*, which was also markedly reduced by vemurafenib and trametinib in SCM containing EGF and bFGF [17], was changed by both drugs in different growth conditions to a similar extent as in SCM (Fig 3a). Secretion of IL-8, which was uniformly downregulated in all tested melanoma cell lines in response to vemurafenib and trametinib in SCM [17], was also reduced by drugs in the medium containing other combinations of growth factors (S5 Fig). *CCND1* expression, which did not significantly differ in untreated melanoma cells grown in the presence of different growth factors (Fig 1b) and was significantly reduced by 10 μM vemurafenib and 50 nM trametinib in cells grown in SCM containing bFGF and EGF [17], was more substantially reduced by trametinib when bFGF was not present in the medium, whereas treatment with vemurafenib induced further reduction of *CCND1* expression only in the medium containing HGF (Fig 3a).

There was no marked differences in alterations induced by vemurafenib and trametinib in activities of ERK1/2, NF-κB and β-catenin assessed in different growth conditions (Fig 3b). Untreated cells grown in the medium containing HGH with either EGF or bFGF exerted a slightly higher level of phosphorylated ERK1/2 (Fig 3b). Next, we have checked the level of endogenous and exogenous HGF in the culture media. A median level of 27.6 ng/ml in the culture medium with recombinant HGF, and of 0.05 ng/ml in the medium containing only endogenous HGF was measured. The level of HGF was a few hundred-fold higher in the medium when exogenous HGF (40 ng/ml) was added in comparison to the medium with HGF released exclusively by melanoma cells (Fig 3c). There was no influence of vemurafenib and trametinib on the HGF level in both, medium with and without exogenous HGF, as shown for DMBC21 melanoma cell population (Fig 3d).

**Fig 4:**
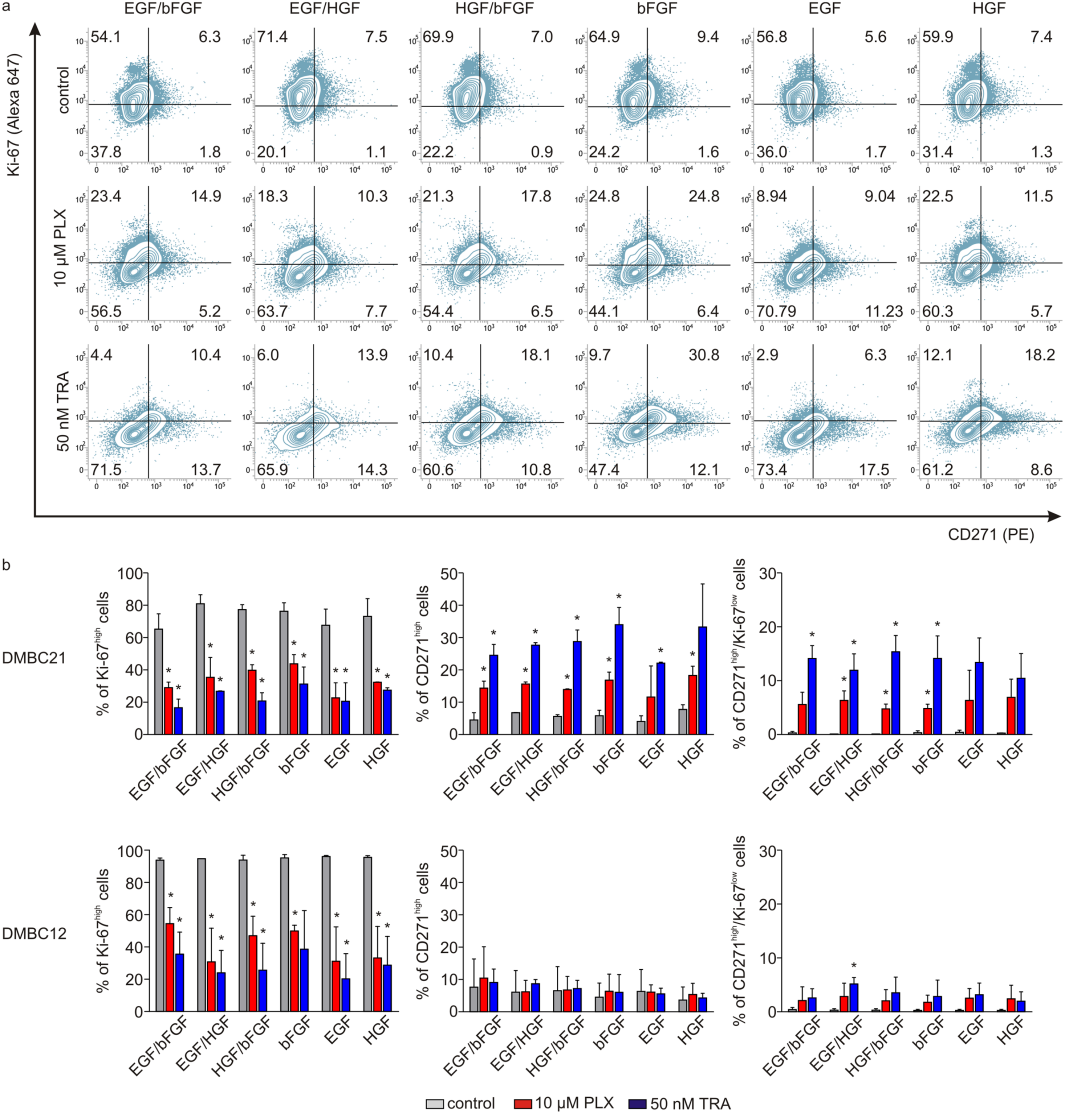
Vemurafenib (PLX) and trametinib (TRA) induce similar changes in the percentages of Ki-67^high^, CD271^high^ and CD271^high^/Ki67^low^ cells in different growth conditions. **a.** Representative flow cytometry contour plots showing changes in the subpopulations of CD271^high^ and Ki-67^high^ cells in DMBC21 melanoma population after treatment with 10 μM PLX and 50 nM TRA for 44 h. Dead cells were excluded from the analysis using the LIVE/DEAD^®^ Fixable Aqua Dead Cell Stain Kit. **b**. Data summarized for DMBC12 and DMBC21 melanoma populations as bar graphs. Data are presented as mean ± SD. Statistical significance of differences (treated vs. control) was calculated with Student's t-test for unmatched samples (α = 0,05). * *P* < 0.05. To analyze the significance of differences in distribution of “stem-like” CD271^high^ and proliferating Ki-67^high^ cells for each treatment among the growth factor groups, Levene's test and Kruskal–Wallis H test were used. No statistically significant differences were found (S1 Table).

Next, we investigated changes in the frequency of CD271^high^ and Ki67^high^ cells after incubation with vemurafenib and trametinib in two cell lines, DMBC12 and DMBC21, in which the expression of these markers in untreated cells differs substantially. We have already demonstrated that vemurafenib and trametinib select CD271^high^ cells representing stem-like melanoma phenotype, while significantly reducing the fraction of Ki-67^high^ fast proliferating cells [17]. In the present study, selection of CD271^high^ cells and reduction of the fraction of Ki67^high^ cells reached similar levels in different media (Fig 4a and 4b). Mutually exclusive expression of CD271 and Ki-67 (CD271^high^/Ki67^low^) was markedly induced by vemurafenib and trametinib in all growth conditions, especially in DMBC21 cell population (Fig 4b).

In general, all above results indicate that there were no differences in drug effects among different growth factor compositions.

### Melanoma cells can grow without exogenous growth factors in the culture medium and respond to targeted therapy

It is known that cancer cells, including melanoma cells, have the capacity to survive with fewer exogenous growth factors than normal cells. Since the influence of different exogenous growth factors on melanoma cell proliferation and cell response to drug treatment was not clearly evident in our study, we asked the question whether providing bFGF, EGF and HGF is necessary at all for ^V600E^BRAF melanoma cell culture propagation, and whether it impacts cell response to vemurafenib and trametinib.

**Fig 5:**
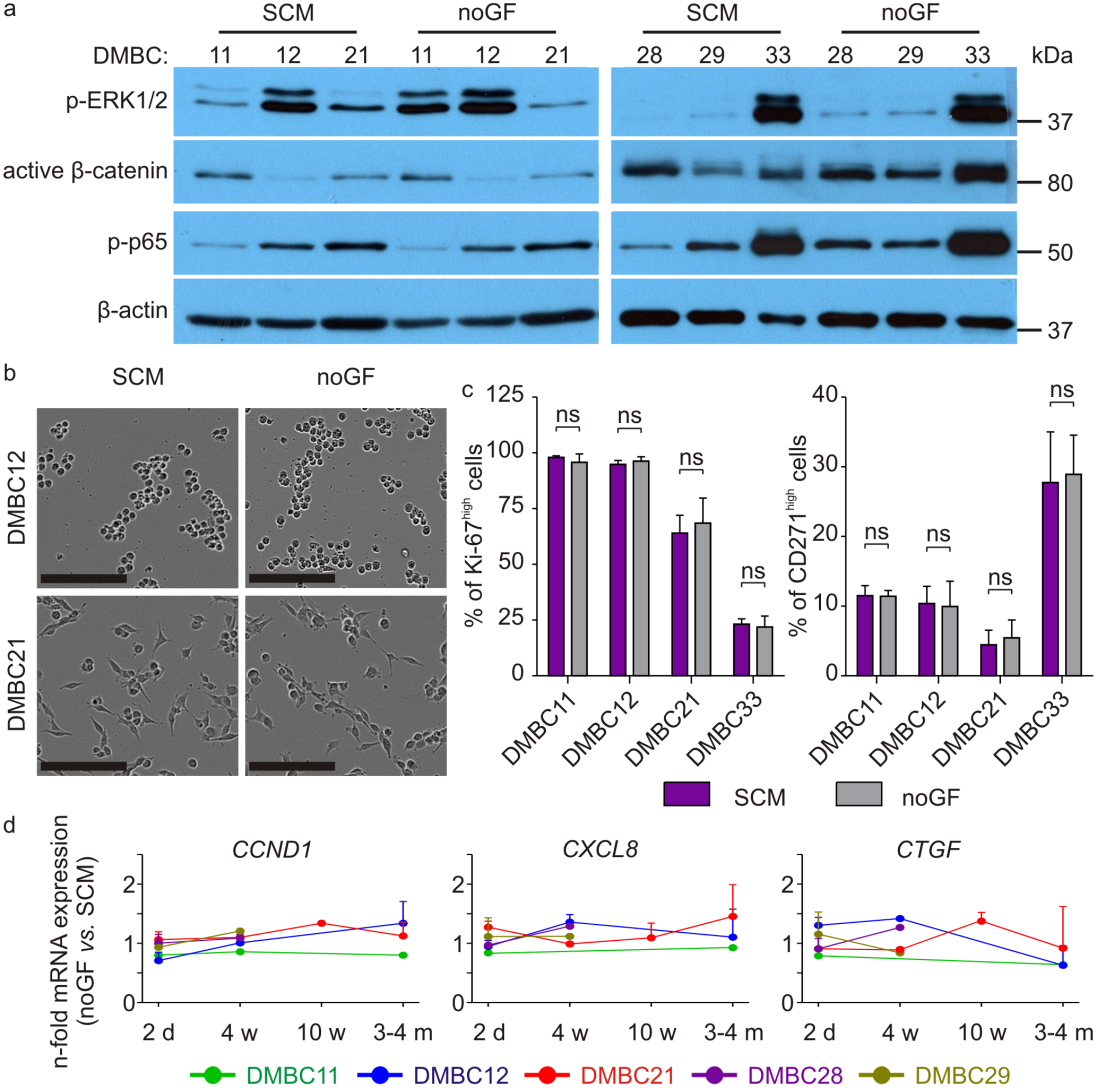
Lack of exogenous growth factors in the culture medium does not markedly affect cell morphology, activity of ERK, β-catenin and NF-κB (p-p65), expression of *CCND1*, *CXCL8* and *CTGF*, and the frequency of Ki-67^high^ and CD271^high^ cells. **a.** Melanoma cells were grown either in SCM or in the medium without these growth factors (noGF) and the levels of phosphorylated ERK1/2 (p-ERK1/2), active β-catenin, and phosphorylated p65 (p-p65) were assessed by immunoblotting. Equal loading was confirmed by β-actin. Representative results are shown. **b.** The microphotographs of cells grown either in SCM or in the medium without growth factors (noGF) for four months (scale bar, 200 μm). They were taken 3 h after cell splitting. **c**. Bar graphs comparing percentages of CD271^high^ and Ki-67^high^ cells in untreated cell populations grown in SCM with percentages of these cells in populations grown in the medium without growth factors (noGF). No statistically significant differences were found (S1 Table). **d**. Basal expression level of *CNND1*, *CXCL8* and *CTGF* was assessed by qRT-PCR and normalized to the expression of a reference gene *RPS17*. Gene expression level in cells grown in the medium without growth factors (noGF) for indicated time (2 days, 4 weeks, 10 weeks, 3–4 months) was expressed relatively to the expression level in cells grown in SCM.

Cell distribution in cell cycle phases was also very similar for melanoma cells grown in SCM containing bFGF and EGF and in the medium without these growth factors for 2 days (S3 Fig). Next, we prolonged the cell culture without growth factors first to 10 days, then to one month, and finally to four months. After 10 days and one month of culturing without growth factors, phenotypes of melanoma cell populations still remained unchanged, as shown for one month in Fig 5a. There was no substantial differences in activities of ERK1/2, NF-κB and β-catenin between cells grown in SCM containing bFGF and EGF and those grown without growth factors as shown for six different melanoma cell populations (Fig 5a) although some variabilities were found between experiments, especially for DMBC11 cells. Lack of growth factors in the culture medium did not substantially affect viability of melanoma cells (not shown), their morphology (Fig 5b) and immunophenotype (Fig 5c and S3 Fig). Thus, the conclusion that melanoma cells can grow without exogenously delivered growth factors bFGF, EGF and HGF might be true for most of melanoma cells. This was further supported by prolonged culturing. No substantial alterations in expression of *CCND1*, *CXCL8* and *CTGF* was observed after 2 days, 4 and 10 weeks and 4 months of culturing without growth factors (Fig 5d).

**Fig 6:**
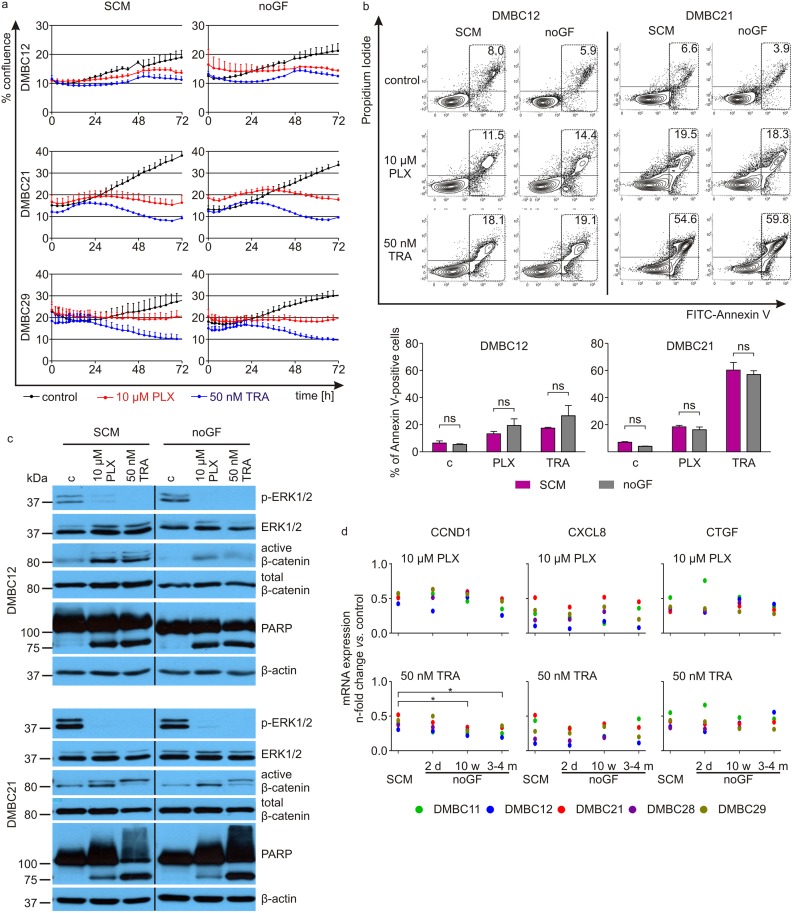
Lack of exogeneous growth factors (bFGF, EGF and HGF) in the culture medium for 4 months does not substantially influence melanoma cell response to vemurafenib and trametinib. **a.** Proliferation time-courses monitored by analyzing the occupied area (% confluence) of cell images using IncuCyte. Effects of 10 μM vemurafenib and 50 nM trametinib on melanoma cells grown before treatment for four months and during treatment either in SCM or in the medium without growth factors (noGF) are shown. **b.** Flow cytometry after Annexin V/propidium iodide staining was used to measure the percentages of apoptotic cells in DMBC12 and DMBC21 cell populations. Typical contour plots and percentages of apoptotic cells (Annexin V-positive) are shown. Bar graphs showing percentages of Annexin V-positive cells in untreated, vemurafenib- and trametinib-treated cell populations. No statistically significant differences were found between drug influence on cells grown in SCM and cells grown in the medium without growth factors (S1 Table). See S3 Fig for results of single experiments performed with DMBC11, DMBC28, DMBC29 and DMBC33 cell populations. **c.** Melanoma cells grown either in SCM or without growth factors (noGF) for 4 months were treated with 10 μM PLX and 50 nM TRA for 44 h and the levels of phosphorylated ERK1/2 (p-ERK1/2), total ERK1/2, active β-catenin, total β-catenin and PARP were assessed by immunoblotting. Equal loading was confirmed by β-actin. Representative results are shown. **d**. qRT-PCR validation of changes in the expression of *CCND1*, *CXCL8* and C*TGF* in melanoma cells after 24 h of incubation with 10 μM PLX and 50 nM TRA relative to the respective control. The expression is normalized to the expression of a reference gene *RPS17*. Points indicate individual values representing a mean from two biological replicates obtained for each of five cell populations (DMBC11, DMBC12, DMBC21, DMBC28, DMBC29). Statistical significance was calculated with ANOVA for repeated measures and Post-Hoc Dunnet test (noGF: 2 days, 10 weeks and 3–4 months) *vs*. SCM, α = 0,05). * *P* < 0.05.

Untreated melanoma cells showed robust proliferation (Fig 6a) and maintained the cell viability very well (Fig 6b and S4 Fig) even when the period of *in vitro* culture without growth factors was extended to 4 months. They responded at the molecular and cellular levels to vemurafenib and trametinib in a similar way as in SCM; the viable cell number was reduced by vemurafenib and trametinib in comparable way (Fig 6a), apoptosis was induced to similar extent as shown by assessment of percentages of Annexin V-positive cells (Fig 6b and S4 Fig) and PARP cleavage (Fig 6c), the activity of β-catenin was enhanced by drugs whereas activity of ERK1/2 was diminished (Fig 6c). The influence of drugs on the expression of *CCND1*, *CXCL8* and *CTGF* was also similar, when impact of drugs on the gene expression was compared between melanoma cells grown in SCM and the medium without growth factors for 2 days, 10 weeks and 3–4 months (Fig 6d). The only exception was the influence of trametinib on *CCND1* expression, as trametinib reduced *CCND1* expression in melanoma cells grown in medium without growth factors for at least 10 weeks to significantly higher extent than in melanoma cells grown in SCM (Fig 6d).

## Discussion

Growth factors are expressed endogenously by cancer cells, and self-sufficiency in growth signals is one of the hallmarks of cancer [22]. It is also evidenced that stromal cells support tumor development. There is no reliable method to quantify a ligand level directly in melanoma microenvironment in a living patient [23], and one can expect that as growth factors are secreted from multiple sources their levels will undergo dynamic changes. Therefore, quantification of ligands in serum is more frequently used. Pretreatment plasma HGF levels from patients enrolled onto the BRIM2 clinical trial ranged from 0.033 ng/ml to 7.2 ng/ml with a median level of 0.33 ng/ml [5]. It has been demonstrated that serum level of HGF significantly correlates with the stage of melanoma [24], although patient evaluation did not provide unambiguous results to validate HGF as a biomarker for melanoma response to BRAF inhibitors [2,5,23–25]. bFGF was identified as an autocrine growth factor for melanoma [26], and blockage of FGFR synergistically enhances effects of BRAF inhibition in melanoma cells [27]. Significantly increased EGF levels were found in sera from patients with melanoma-positive sentinel lymph nodes as compared with sera from sentinel lymph node-negative patients, and it has been demonstrated that EGF facilities melanoma lymph node metastasis [28]. EGFR inhibitors cooperated with BRAF inhibitors to block the growth of BRAF inhibitor-resistant cells *in vitro* and *in vivo* [29]. Interestingly, it has been demonstrated that targeting ^V600E^BRAF and MEK1/2 with inhibitors leading to ERK1/2 inhibition causes a relief of ERK-dependent feedback and reactivation of ligand-dependent signal transduction in melanoma and stroma [1–3,5].

A strategy employed in this study was to substitute paracrine contribution from melanoma stroma with exogenous bFGF, EGF and HGF, provided either alone or in combination in the culture medium, and then extensively investigate the possible alteration in melanoma cell phenotype and cell response to vemurafenib and trametinib. Alternatively, we assessed the same parameters while culturing melanoma cells in the medium lacking exogenous bFGF, EGF and HGF for about 4 months, leaving cells dependent exclusively on autocrine growth factor production. Our study did not reveal any substantial influence of these growth factors on melanoma viability and phenotype. Effects of vemurafenib and trametinib on viability, cell cycle, percentages of Ki-67^high^ (intensively proliferating) and CD271^high^ (stem-like) melanoma cells and activity of signaling pathways crucial for melanoma development and maintenance, including the RAF/MEK/ERK pathway, WNT/β-catenin pathway and NF-κB signaling were very similar irrespective of growth conditions, at least within the first 72 h of treatment. We cannot exclude that growth factor-dependent signal transduction become hyper-activated later in the development of resistance. Gene expression of *CXCL8*, *CCND1*, *DKK1* and *CTGF* were similarly changed by drugs irrespective of growth conditions. Expression of *CCND1*, which was significantly reduced by trametinib and vemurafenib [17], was even more efficiently reduced by trametinib in the medium lacking exogenous bFGF. This, however, did not result in higher accumulation of cells in G_1_ phase of cell cycle, indicating that although significant differences were observed in the level of cyclin D1 transcript, they were not high enough to impact cell distribution in the cell cycle. In ^V600E^BRAF melanoma cells cyclin D1 is constitutively expressed. Its induction is growth factor-independent and mainly dependent on MEK activation and nuclear accumulation of phosphorylated ERK [30]. This explains why its expression is substantially reduced by vemurafenib and trametinib. Additional pathways, however, might also be involved in *CCND1* expression as shown in other cell types [31–33], which might contribute to the observed small differences in cyclin D1 transcript levels when drugs were used in the presence of different growth signals.

While growth factor independence of ^V600E^BRAF melanoma cells was not surprising and was already observed that a ligand stimulation had little or no effect on proliferation of kinase-addicted cells [5], lack of influence of growth factors on cell response to vemurafenib and trametinib was intriguing in the light of previously published results. It has been demonstrated that the stroma-mediated influence of targeted therapy on upstream elements of targeted pathways results in diminution of the drug effectiveness [1,2,5]. Careful examination of the *in vitro* results reported in these studies revealed that this phenomenon was not universal. EGF, HGF and FGF strongly antagonized the vemurafenib sensitivity only in some of ^V600E^BRAF melanomas tested; HGF in 5, EGF in 3 and different FGFs in not more than 1 out of 8 melanoma cell lines [1]. A marked influence of recombinant HGF (0.25–50 ng/ml) on the vemurafenib analog (PLX4720)-inhibited proliferation was observed only in one (SK-MEL-5) out of 3 melanoma cell lines [2]. In another study, one out of seven melanoma cell lines could be rescued from vemurafenib-induced growth inhibition by either HGF or FGF, but none by EGF, and in other cell lines only ‘partial rescue’ was observed [5]. Among 12 additional ^V600E^BRAF melanoma cell lines, HGF significantly attenuated vemurafenib sensitivity only in 5 lines [5]. Moreover, correlation between cMET expression and HGF rescue in vemurafenib-treated BRAF-mutant melanoma cells was not high in this study (*r*^*2*^ = 0.56) suggesting that cMET contribution to microenvironment-mediated BRAF-inhibitor resistance remains to be clarified. More recently it was demonstrated that the cMET clinical score was not a statistically significant predictive factor for therapy with BRAF-inhibitor [25], and stromal or parenchymal HGF expression levels did not indicate tumor response to inhibitors of BRAF [23]. Although, the ligand-induced activity of receptors was not investigated in the present study, there is a low probability that none of the receptors could be activated by tested growth factors in all six patient-derived samples.

The discrepancies between the *in vitro* results obtained by different laboratories might be due to different experimental conditions. Moreover, differences in the response to ^V600E^BRAF and MEK1/2 inhibitors *in vitro* might also reflect the variability in the degree of the response observed among the ^V600E^BRAF melanoma patients. More experiments, especially in patient-derived melanoma cell populations closely mimicking the original tumors, should be performed. Alternatively, more reliable methods to measure the levels of growth factors released by melanoma cells and stromal cells in patients’ tumors might be developed to investigate their potential role in defining patients with poor response to BRAF/MEK inhibitor therapy.

Another interesting aspect of our study is that it raised the question about the necessity of using bFGF, EGF and HGF in culture medium for *in vitro* investigation of melanoma biology and melanoma response to drugs targeting the RAF/MEK/ERK pathway in ^V600E^BRAF melanoma cells. It would be interesting to find out whether the medium without growth factors can be sufficient to establish cell lines from surgery specimens, and whether the tumor heterogeneity and molecular characteristics of original tumor could be kept unaffected to a large extent in these conditions.

## Supporting information

S1 Fig Composition of exogenous growth factors (bFGF, HGF and EGF) does not substantially affect vemurafenib (PLX)- and trametinib (TRA)-induced apoptosis in DMBC28 cell population. Drugs were used at two concentrations, PLX at 3 μM and 10 μM and TRA at 20 nM and 50 nM. Percent of apoptotic cells with high caspase 3/7 activity was assessed in time-lapse imaging system IncuCyte over the course of 72 h. Apoptotic response was not induced in any growth conditions at lower concentrations of drugs during 72 h of incubation. Therefore, higher concentrations of drugs were used in the study.
(TIF)

S2 Fig Cell cycle profiles of melanoma cells DMBC12 and DMBC33 were determined by flow cytometry. Representative histograms and their quantification from a representative experiment are shown. ModFit LT 3.0 software was used to calculate the percentages of viable cells in cell cycle phases.
(TIF)

S3 Fig Lack of growth factors in the culture medium does not influence cell distribution in cell cycle phases and the percentages of CD271^high^ and Ki-67^high^ cells. **a.** Cell cycle profiles of DMBC11, DMBC12, DMBC21 and DMBC33 cell populations grown in SCM containing bFGF and EGF and in the medium without these growth factors for 2 days were determined by flow cytometry. Representative histograms and their quantification are shown. ModFit LT 3.0 software was used to calculate the percentages of viable cells in cell cycle phases. **b**. Representative flow cytometry contour plots showing percentage of CD271^high^ and Ki-67^high^ cells in DMBC11, DMBC12, DMBC21 and DMBC33 melanoma populations grown either in SCM and in the medium without growth factors (noGF) for 10 days. Dead cells were excluded from the analysis using the LIVE/DEAD^®^ Fixable Aqua Dead Cell Stain Kit. **c**. Bar graphs comparing percentages of CD271^high^ and Ki-67^high^ cells in the populations grown in SCM with percentages of these cells in populations grown in the medium without growth factors (noGF) for indicated time (2 days, 10 days, 4 months).
(TIF)

S4 Fig Lack of exogeneous growth factors (bFGF, EGF and HGF) in the culture medium for 4 months does not substantially influence apoptotic response of DMBC11, DMBC28, DMBC29 and DMBC33 cells to vemurafenib and trametinib. Flow cytometry after Annexin V/propidium iodide staining was used to measure the percentages of apoptotic cells. Typical contour plots and average percentages of apoptotic cells (Annexin V-positive) are shown.
(TIF)

S5 Fig IL-8 secretion by DMBC12 cells grown in SCM containing bFGF and EGF and in the presence of HGF alone and in combination with different growth factors. ELISA was used to assess IL-8 secretion in culture medium collected after 24 h of incubation with indicated drug. Data are presented as fold change in drug-treated cultures *versus* control culture, in which the secretion level of IL-8 was set as 1. The mean values and SD were calculated from at least 2 experiments.
(TIF)

S6 Fig The scans of original WB blots from which the figure panels were made. (PDF)

S1 Table Results of statistical analysis. (DOCX)
