# Supplementary figures and images for: Exogenous growth factors bFGF, EGF and HGF do not influence viability and phenotype of V600EBRAF melanoma cells and their response to vemurafenib and trametinib in vitro
Source: PLoS One. 2017 Aug 22;12(8):e0183498. doi: 10.1371/journal.pone.0183498 (PMC5568748; doi:10.1371/journal.pone.0183498)

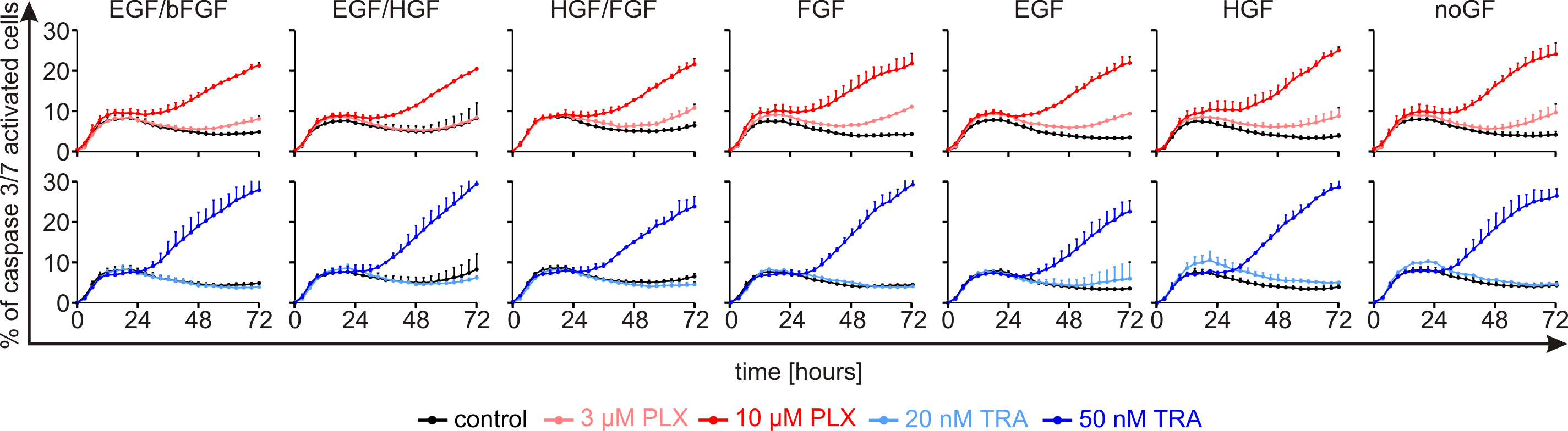

Supplement: S1 Fig — Drugs were used at two concentrations, PLX at 3 μM and 10 μM and TRA at 20 nM and 50 nM. Percent of apoptotic cells with high caspase 3/7 activity was assessed in time-lapse imaging system IncuCyte over the course of 72 h. Apoptotic response was not induced in any growth conditions at lower concentrations of drugs during 72 h of incubation. Therefore, higher concentrations of drugs were used in the study. (TIF) [file pone.0183498.s001.TIF]

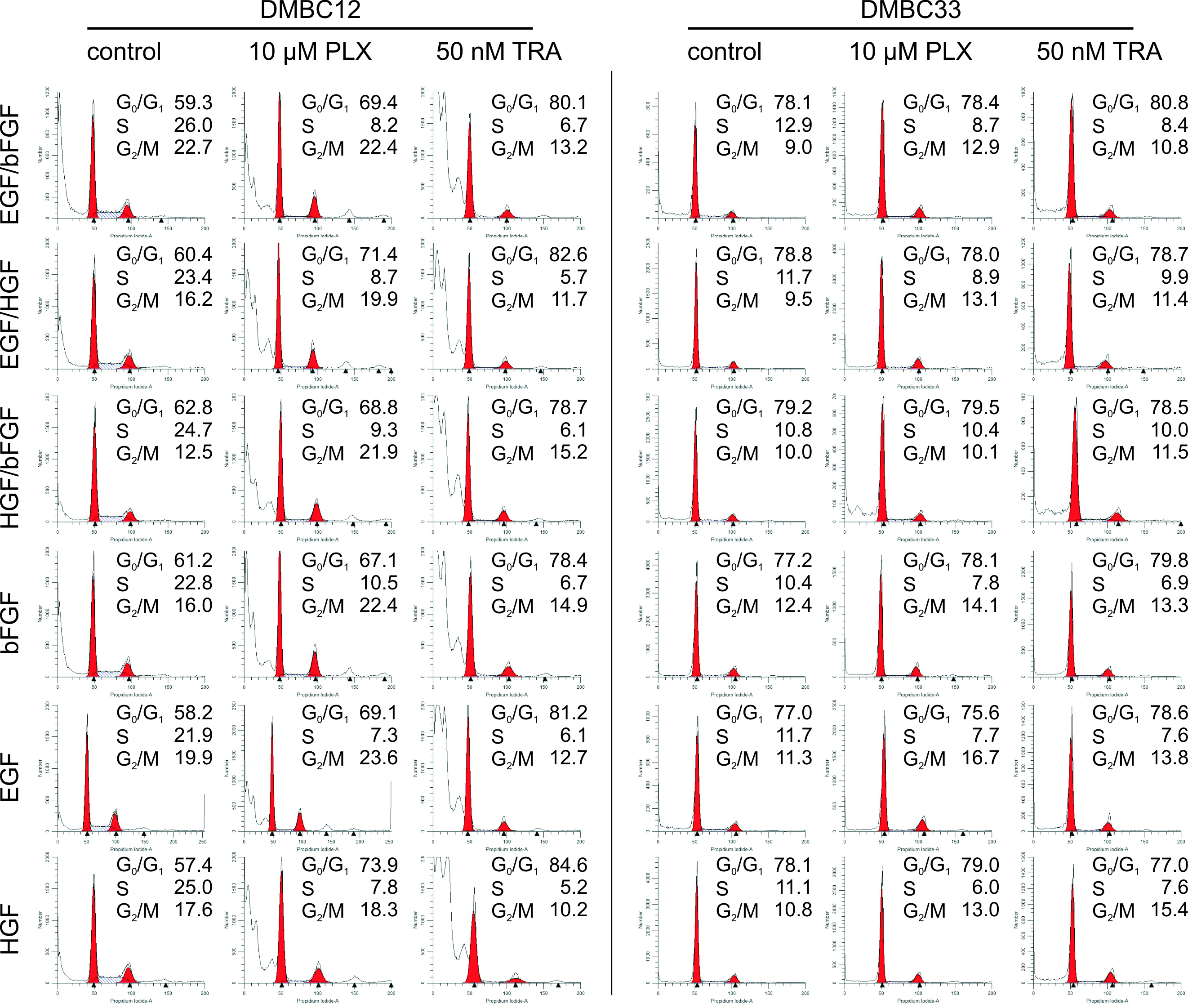

Supplement: S2 Fig — Representative histograms and their quantification from a representative experiment are shown. ModFit LT 3.0 software was used to calculate the percentages of viable cells in cell cycle phases. (TIF) [file pone.0183498.s002.TIF]

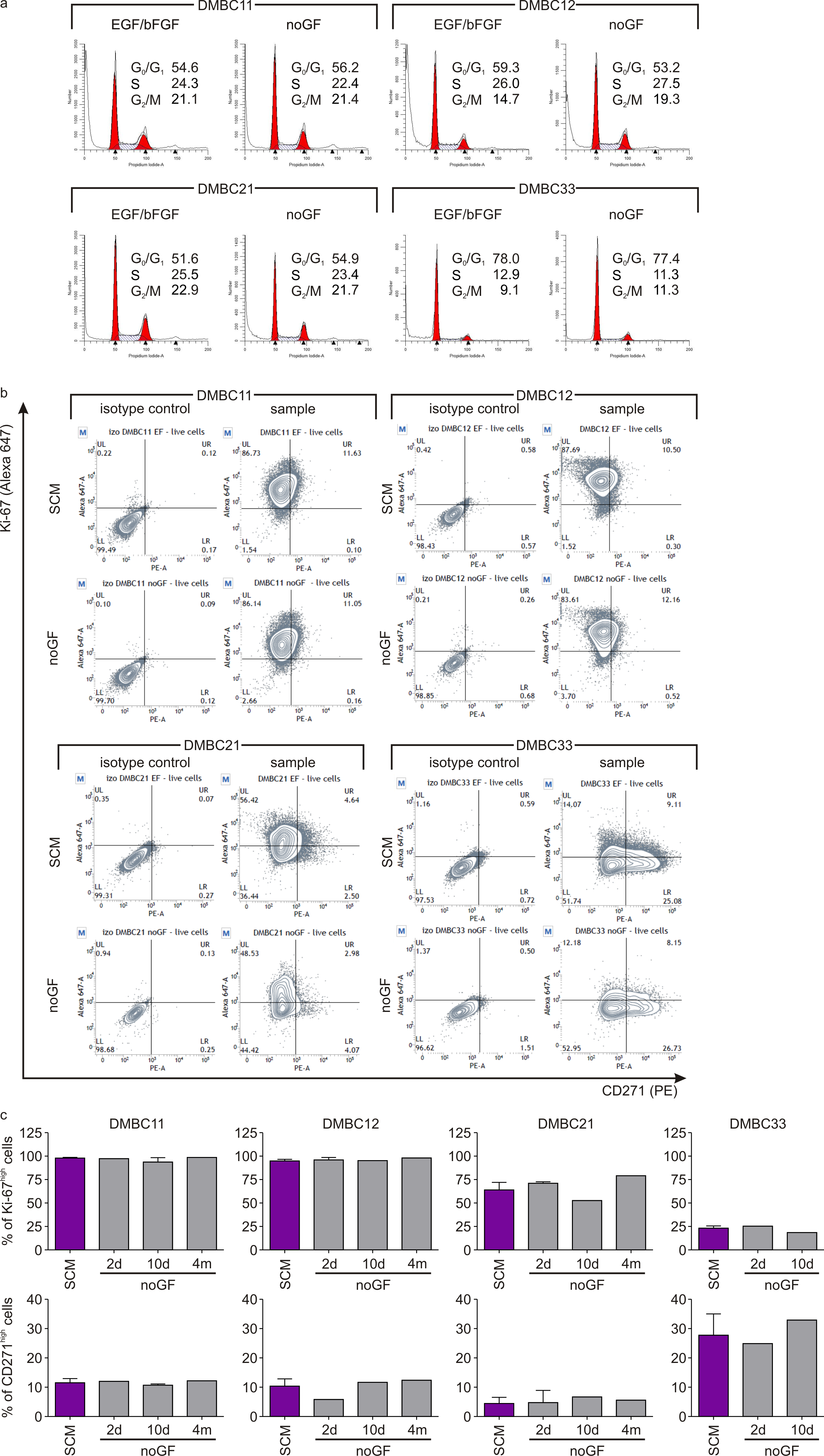

Supplement: S3 Fig — a. Cell cycle profiles of DMBC11, DMBC12, DMBC21 and DMBC33 cell populations grown in SCM containing bFGF and EGF and in the medium without these growth factors for 2 days were determined by flow cytometry. Representative histograms and their quantification are shown. ModFit LT 3.0 software was used to calculate the percentages of viable cells in cell cycle phases. b. Representative flow cytometry contour plots showing percentage of CD271high and Ki-67high cells in DMBC11, DMBC12, DMBC21 and DMBC33 melanoma populations grown either in SCM and in the medium without growth factors (noGF) for 10 days. Dead cells were excluded from the analysis using the LIVE/DEAD® Fixable Aqua Dead Cell Stain Kit. c. Bar graphs comparing percentages of CD271high and Ki-67high cells in the populations grown in SCM with percentages of these cells in populations grown in the medium without growth factors (noGF) for indicated time (2 days, 10 days, 4 months). (TIF) [file pone.0183498.s003.TIF]

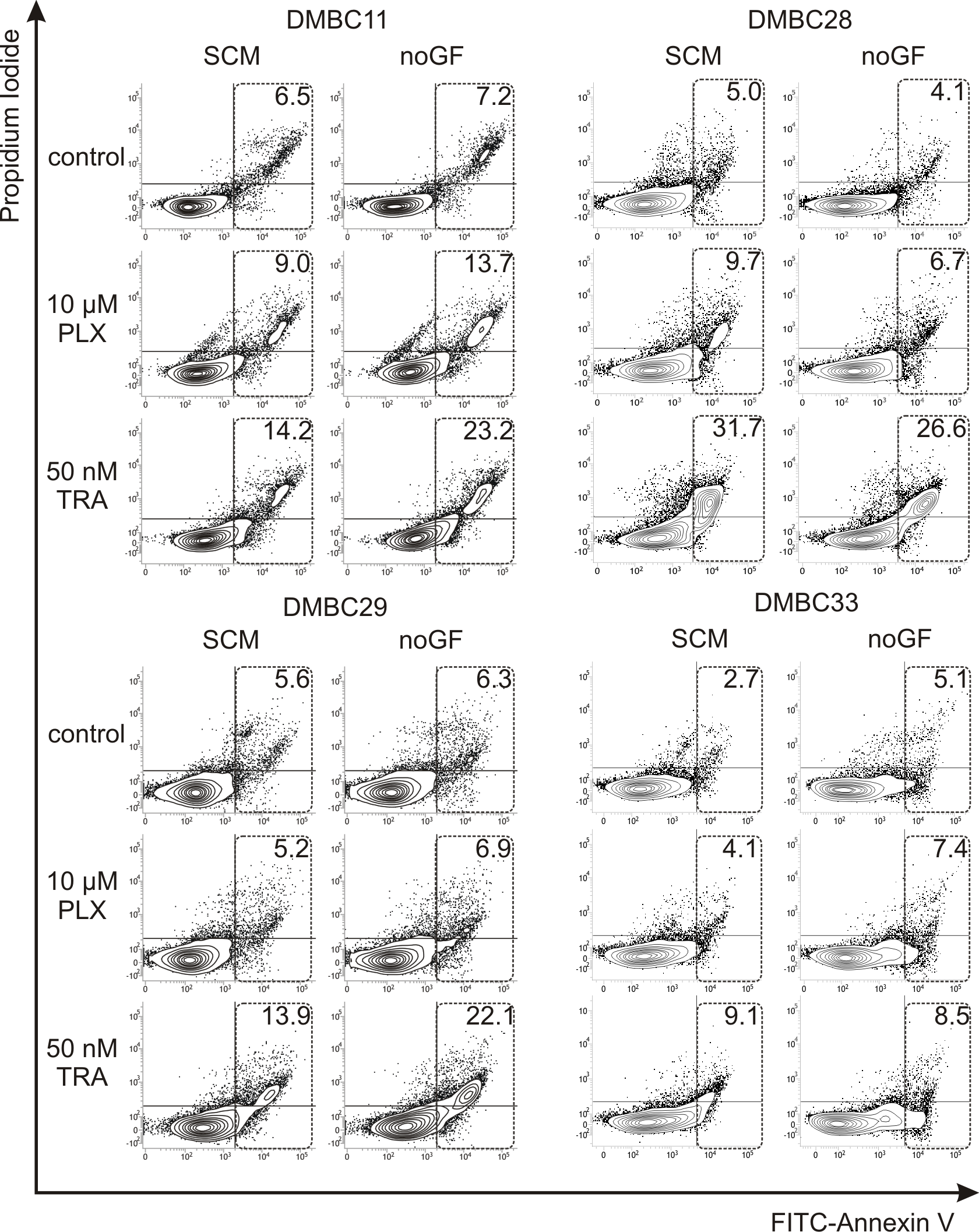

Supplement: S4 Fig — Flow cytometry after Annexin V/propidium iodide staining was used to measure the percentages of apoptotic cells. Typical contour plots and average percentages of apoptotic cells (Annexin V-positive) are shown. (TIF) [file pone.0183498.s004.TIF]

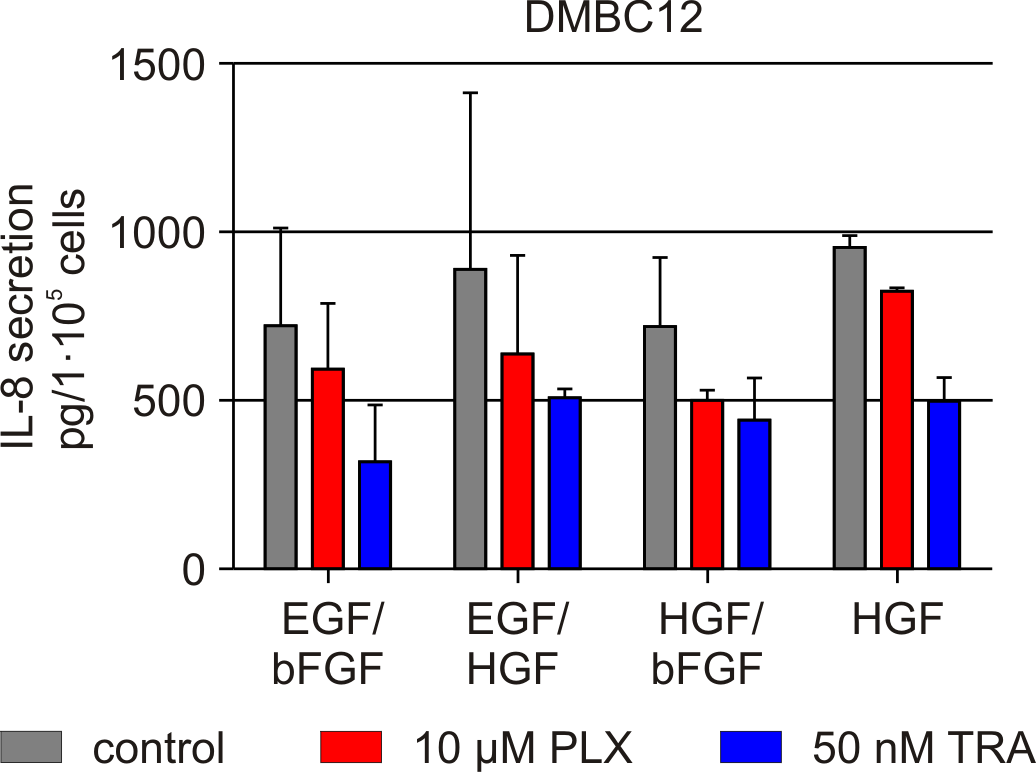

Supplement: S5 Fig — ELISA was used to assess IL-8 secretion in culture medium collected after 24 h of incubation with indicated drug. Data are presented as fold change in drug-treated cultures versus control culture, in which the secretion level of IL-8 was set as 1. The mean values and SD were calculated from at least 2 experiments. (TIF) [file pone.0183498.s005.TIF]
