# Supplementary material for: Exogenous growth factors bFGF, EGF and HGF do not influence viability and phenotype of V600EBRAF melanoma cells and their response to vemurafenib and trametinib in vitro
Source: PLoS One. 2017 Aug 22;12(8):e0183498. doi: 10.1371/journal.pone.0183498 (PMC5568748; doi:10.1371/journal.pone.0183498)
Supplement: S6 Fig — (PDF) [file pone.0183498.s006.pdf]

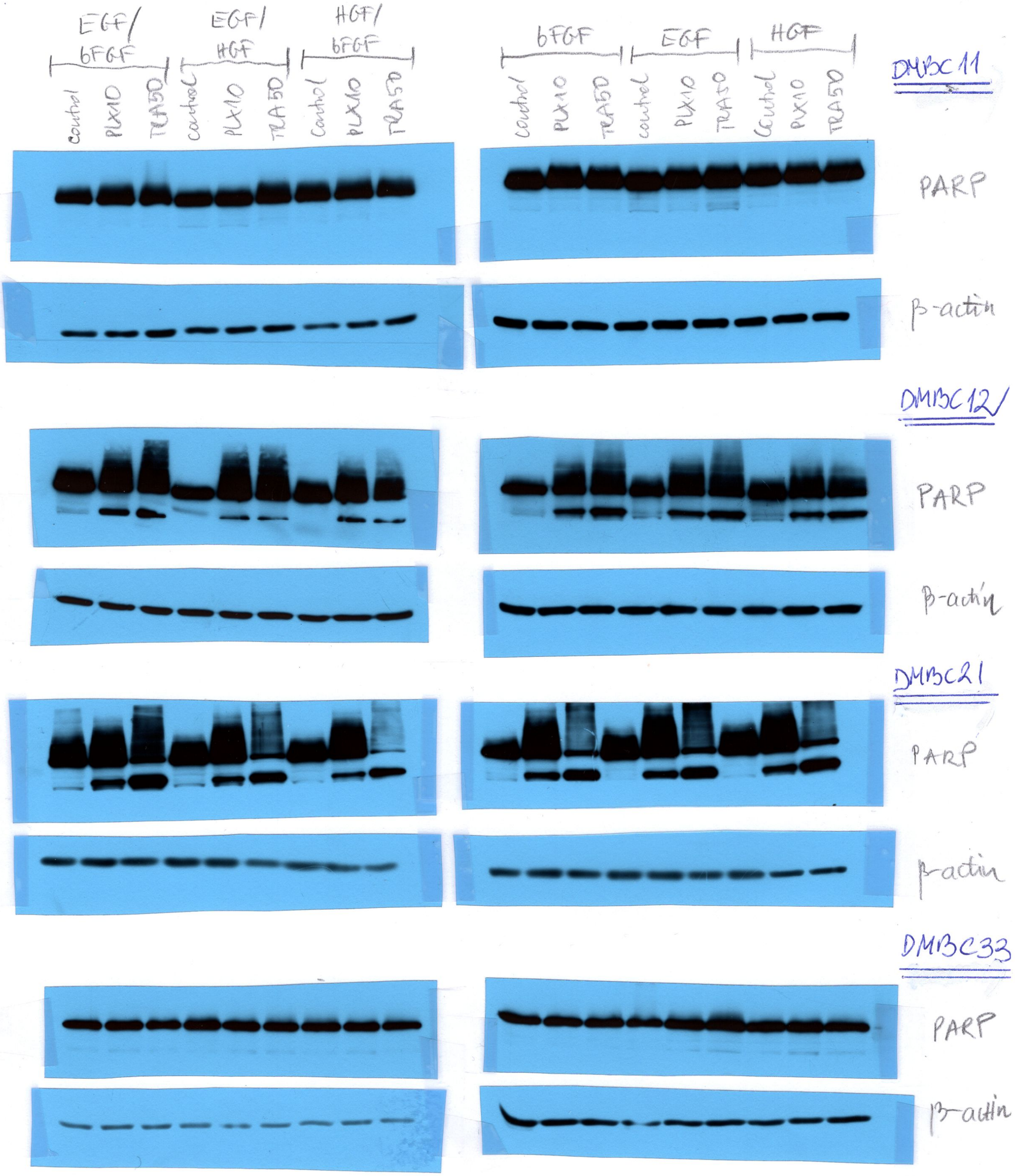
EGF/bFGF
EGF/HGF
HGF/bFGF
bFGF
EGF
HGF
control
PLX10
TRA50
DMBC11
PARP
β-actin
DMBC12
DMBC21
DMBC33

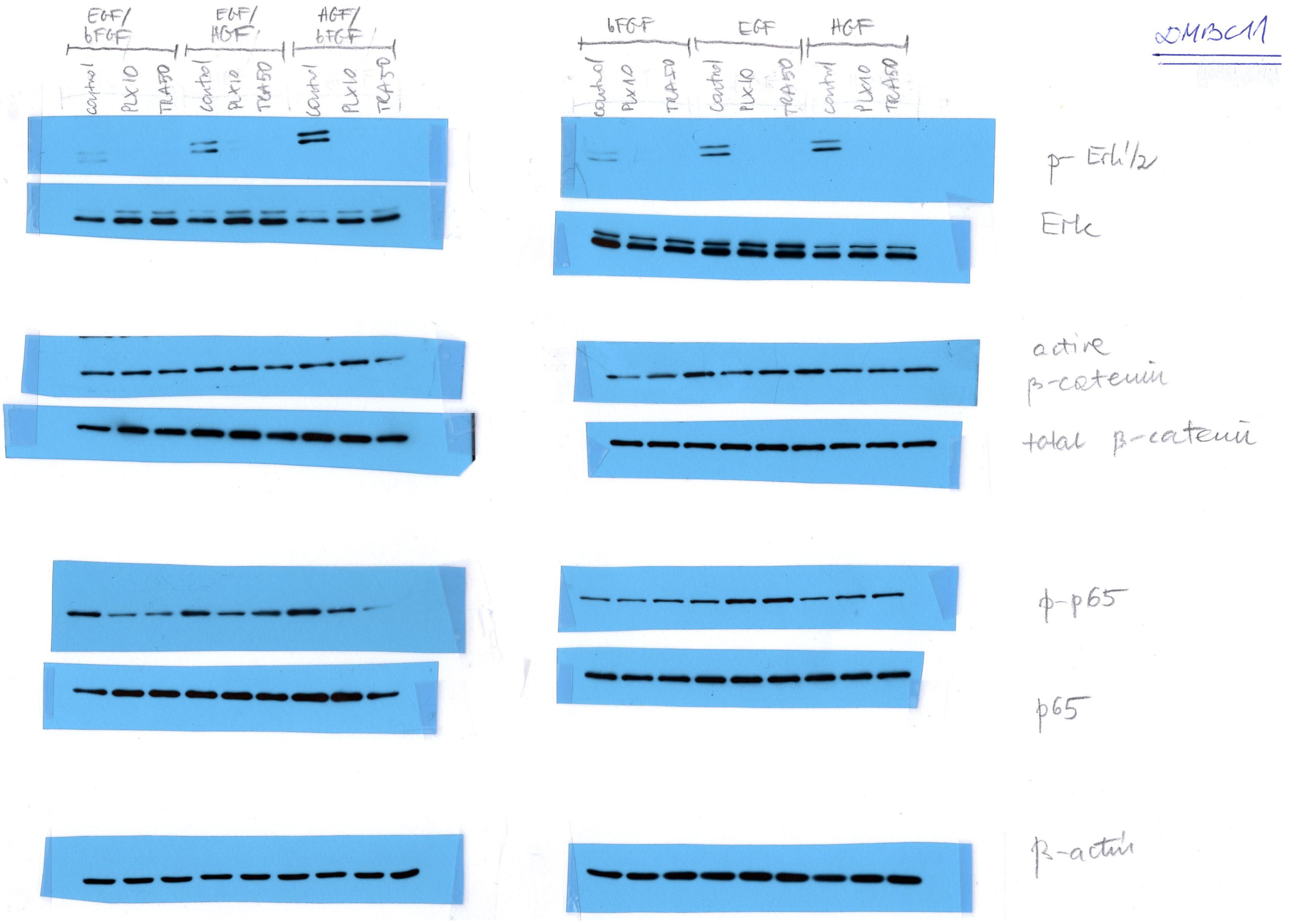

EGF/bFGF
EGF/HGF
HGF/bFGF
control
PLX10
TRA50
bFGF
EGF
HGF
p-Erk1/2
Erk
active β-catenin
total β-catenin
p-p65
p65
β-actin

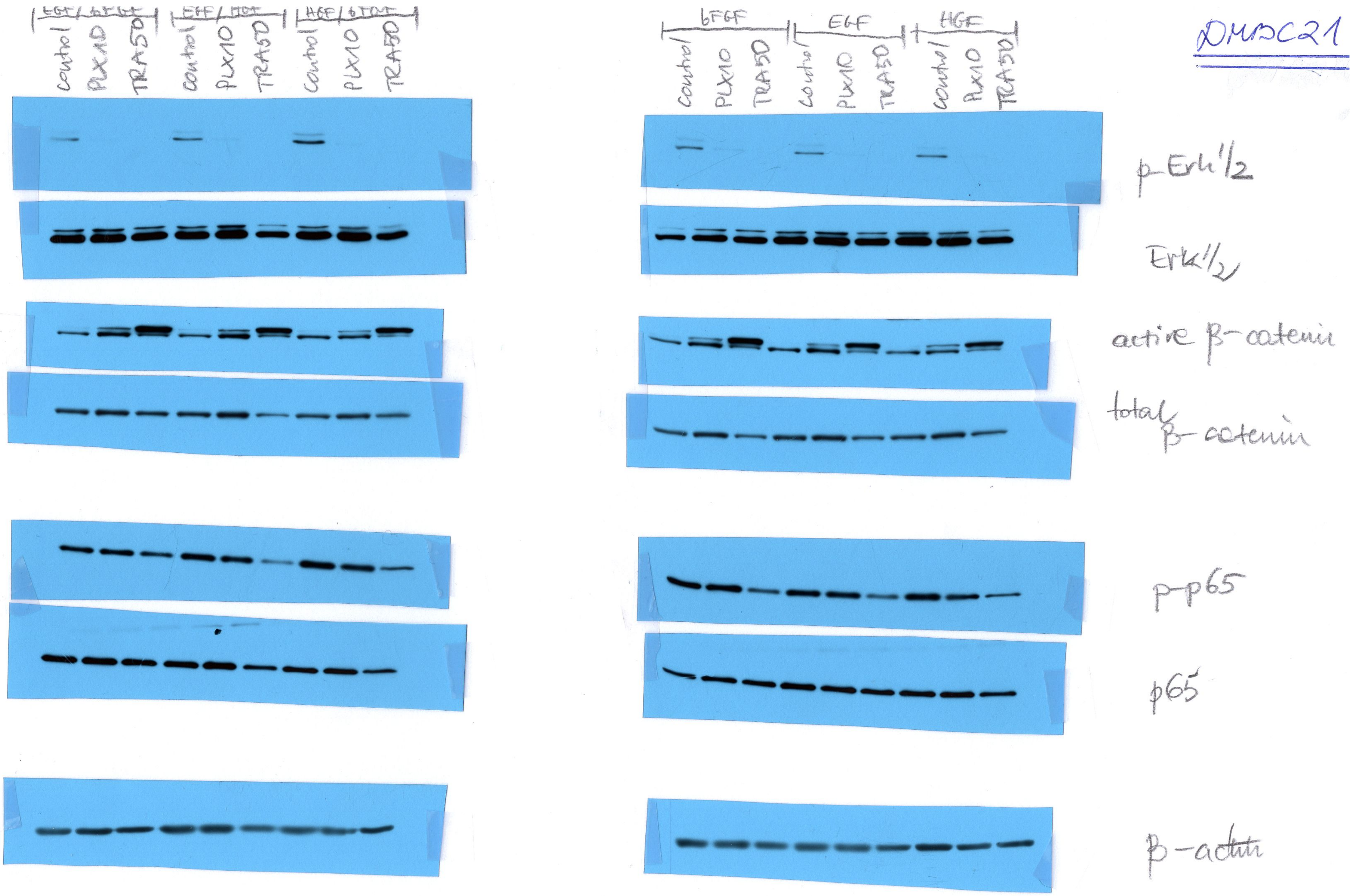

EGF / bFGF
control
PLX10
TRA50
EGF / HGF
control
PLX10
TRA50
HGF / bFGF
control
PLX10
TRA50
bFGF
control
PLX10
TRA50
EGF
control
PLX10
TRA50
HGF
control
PLX10
TRA50
DMBC21
p-Erk1/2
Erk1/2
active β-catenin
total β-catenin
p-p65
p65
β-actin

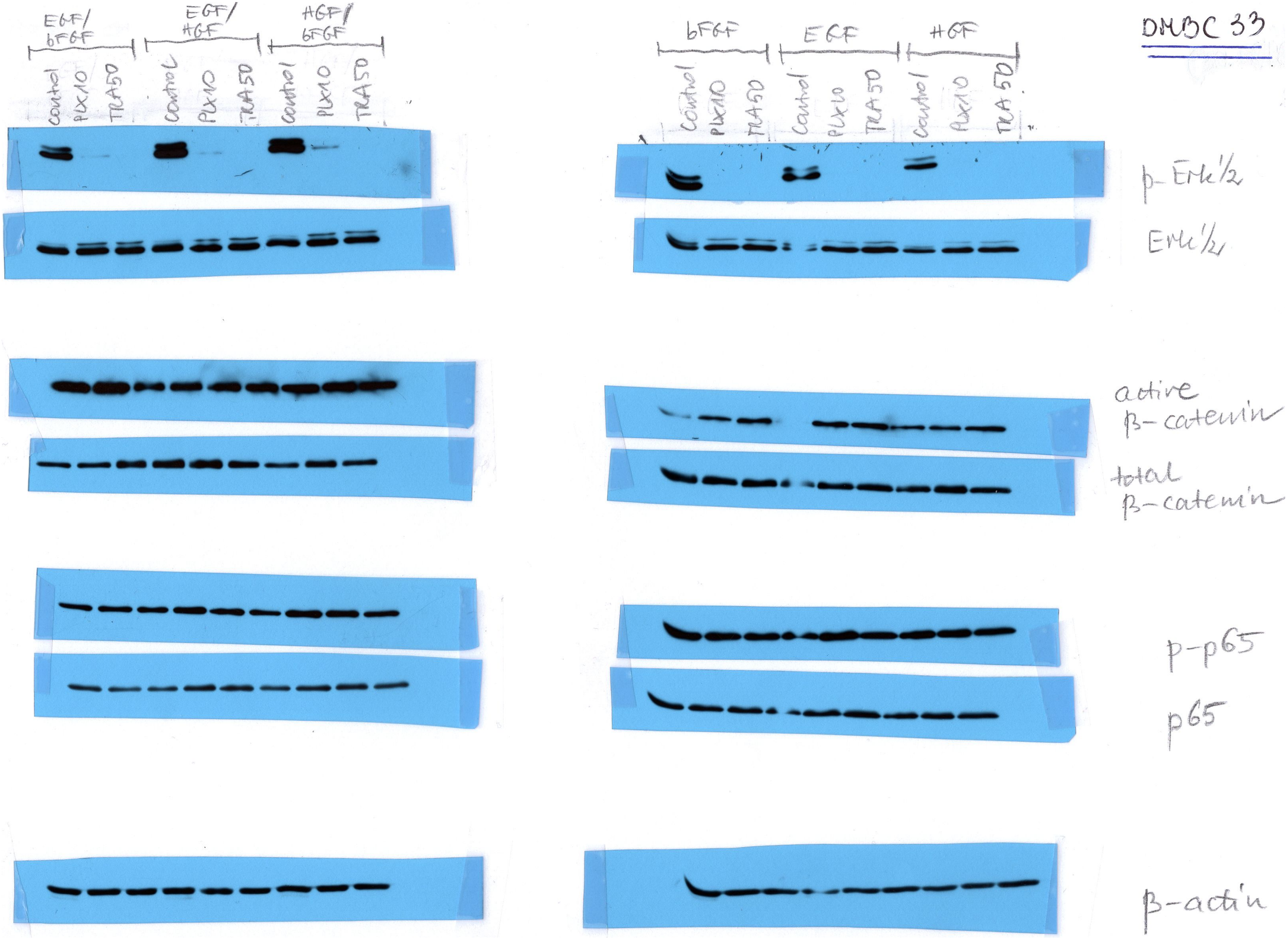
DMBC 33
EGF/ bFGF
EGF/ HGF
HGF/ bFGF
control
PLX10
TRA50
bFGF
EGF
HGF
p-Erk1/2
Erk1/2
active β-catenin
total β-catenin
p-p65
p65
β-actin

DMBC11-21

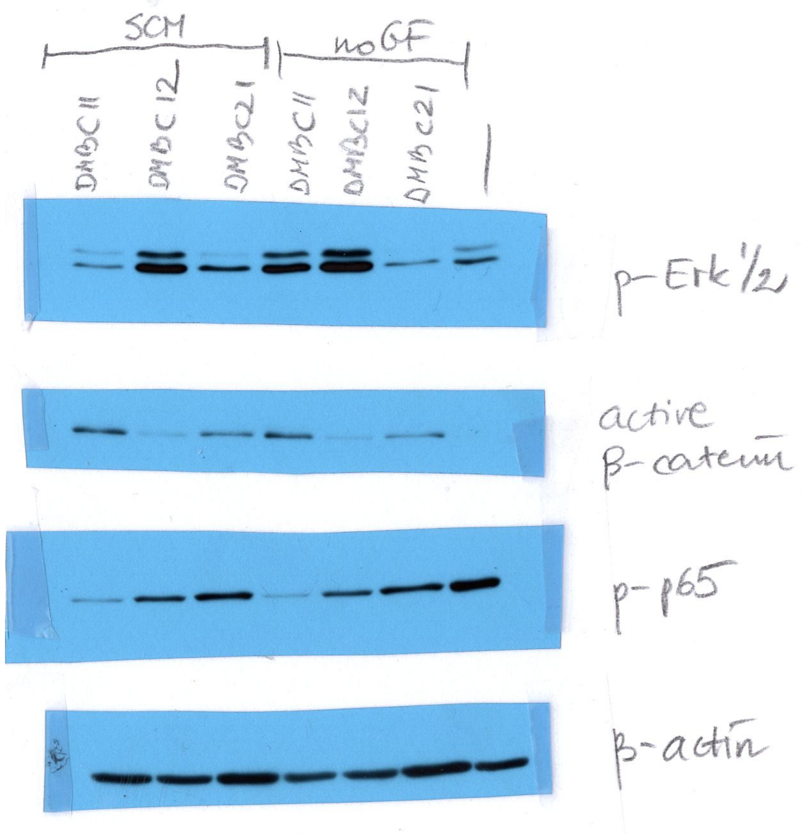

DMBC28-33

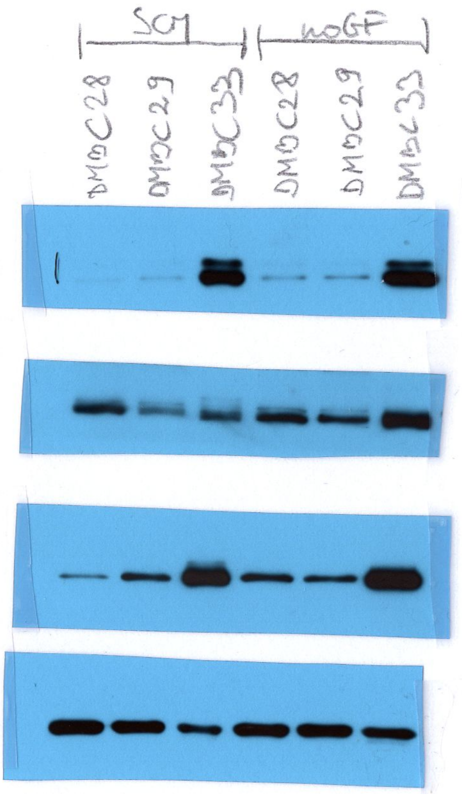

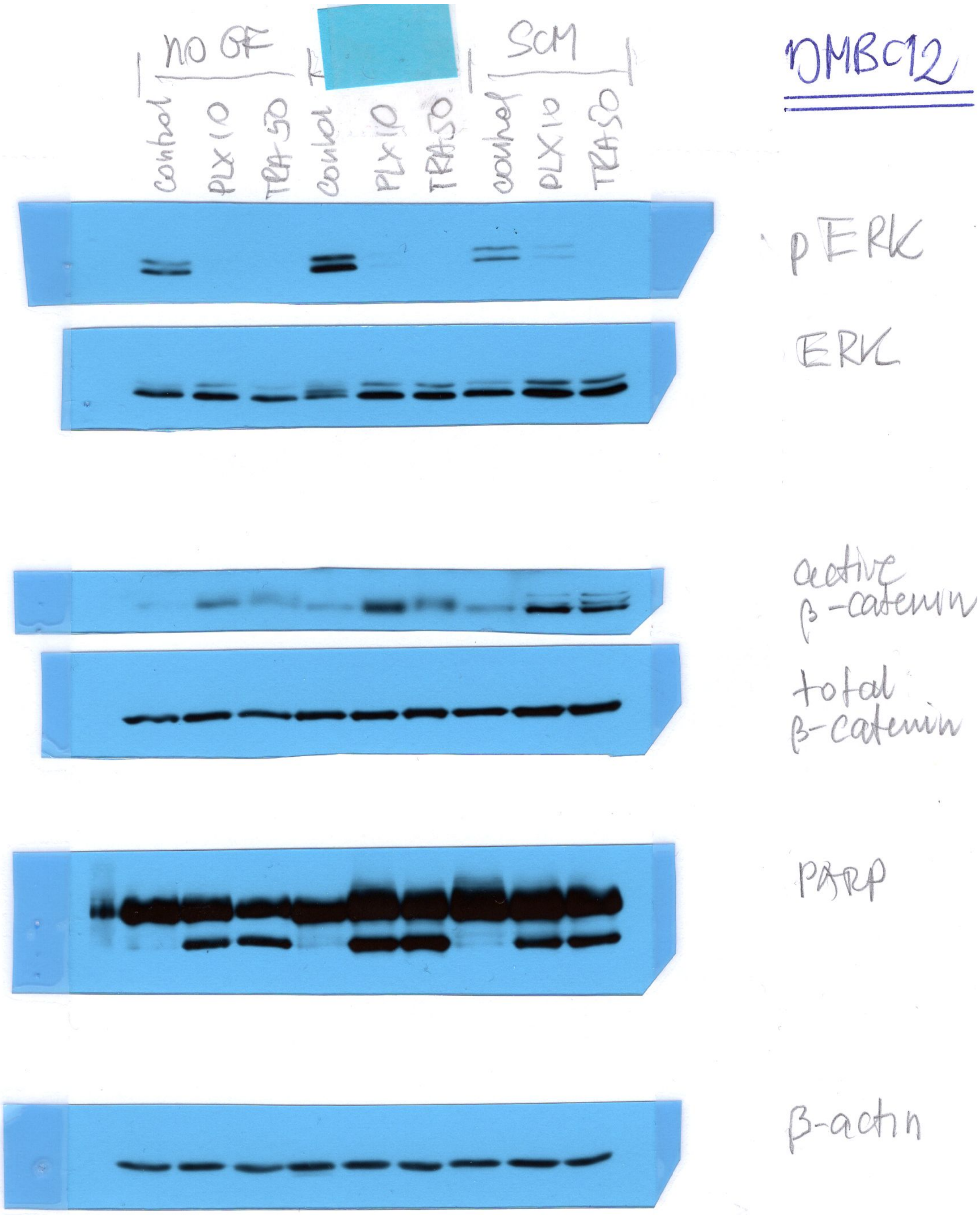
DMBC12
no GF
SCM
control
PLX10
TRA50
control
PLX10
TRA50
control
PLX10
TRA50
pERK
ERK
active β-catenin
total β-catenin
PARP
β-actin

DMBC21

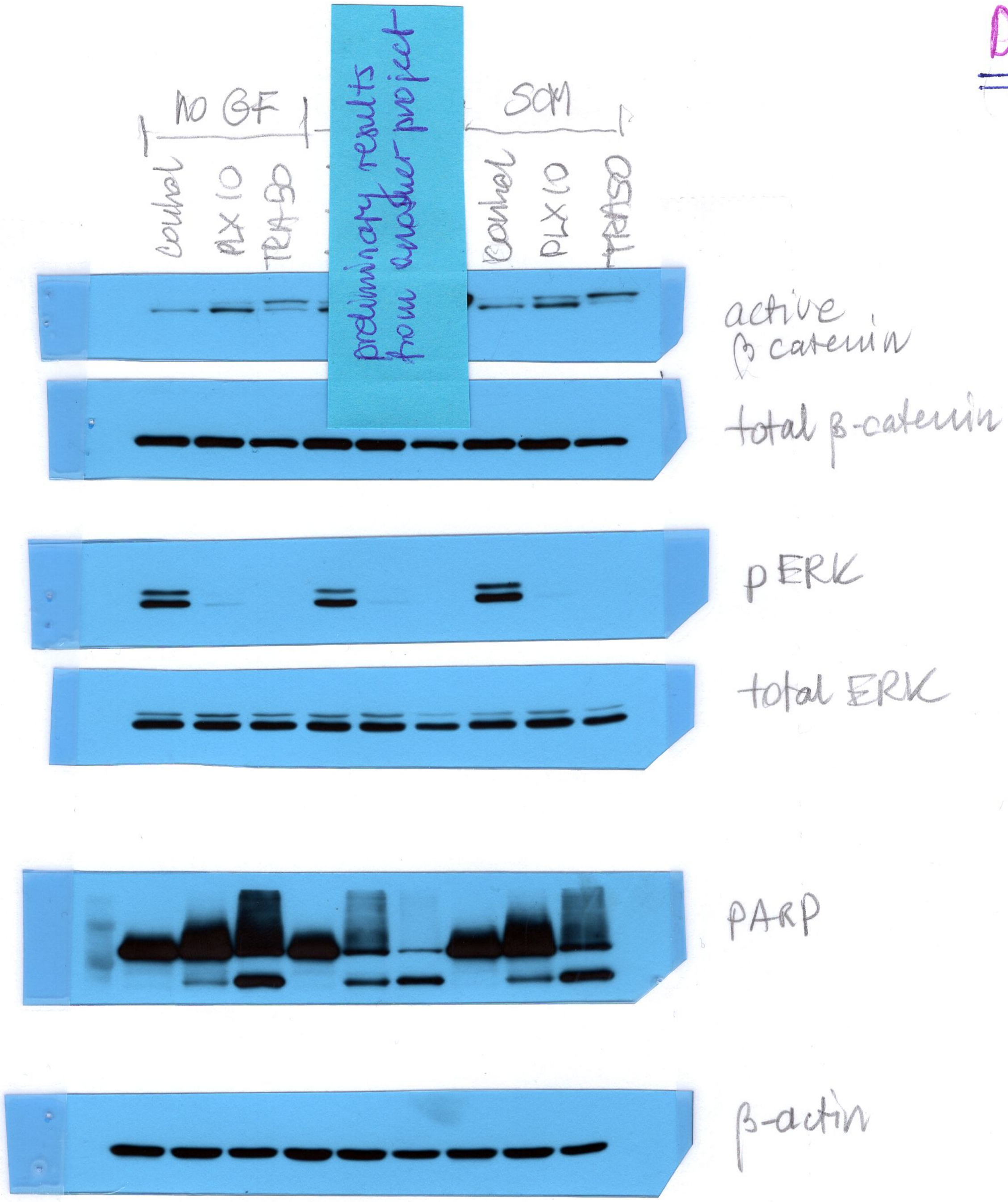
no GF
SCM
control
PLX10
TRA50
preliminary results from another project
control
PLX10
TRA50
active β catenin
total β-catenin
pERK
total ERK
PARP
β-actin
