# Supplementary material for: Exogenous growth factors bFGF, EGF and HGF do not influence viability and phenotype of V600EBRAF melanoma cells and their response to vemurafenib and trametinib in vitro
Source: PLoS One. 2017 Aug 22;12(8):e0183498. doi: 10.1371/journal.pone.0183498 (PMC5568748; doi:10.1371/journal.pone.0183498)
Supplement: S1 Table — (DOCX) [file pone.0183498.s007.docx]

| **Cell line** | **n** | **Levene's test result (p)** | **ANOVA result (p)** | **Kruskal-Wallis H test result (p)** |
| --- | --- | --- | --- | --- |
| DMBC11 | 2 | <0,0001 | - | 0,9548 |
| DMBC12 | 3 | 0,7608 | 0,2038 | - |
| DMBC21 | 2 | <0,0001 | - | 0,8871 |

Fig 1b:

Fig 4b:

| **Cell line** | **Treatment** | **Antigen** | **Levene's test result (p)** | **Kruskal-Wallis H test result (p)** |
| --- | --- | --- | --- | --- |
| DMBC12 | C | Ki-67+ | <0,0001 | 0,5084 |
| DMBC12 | C | CD271+ | <0,0001 | 0,8717 |
| DMBC12 | C | CD271+/Ki-67- | <0,0001 | 0,9845 |
| DMBC12 | PLX | Ki-67+ | <0,0001 | 0,2573 |
| DMBC12 | PLX | CD271+ | <0,0001 | 0,9834 |
| DMBC12 | PLX | CD271+/Ki-67- | <0,0001 | 0,9316 |
| DMBC12 | TRA | Ki-67+ | <0,0001 | 0,7707 |
| DMBC12 | TRA | CD271+ | <0,0001 | 0,5167 |
| DMBC12 | TRA | CD271+/Ki-67- | <0,0001 | 0,5955 |
| DMBC21 | C | Ki-67+ | <0,0001 | 0,3215 |
| DMBC21 | C | CD271+ | <0,0001 | 0,2842 |
| DMBC21 | C | CD271+/Ki-67- | <0,0001 | 0,5747 |
| DMBC21 | PLX | Ki-67+ | <0,0001 | 0,1987 |
| DMBC21 | PLX | CD271+ | <0,0001 | 0,4159 |
| DMBC21 | PLX | CD271+/Ki-67- | <0,0001 | 0,9470 |
| DMBC21 | TRA | Ki-67+ | <0,0001 | 0,4955 |
| DMBC21 | TRA | CD271+ | <0,0001 | 0,2509 |
| DMBC21 | TRA | CD271+/Ki-67- | <0,0001 | 0,7823 |

Fig 5c:

| **Cell line** | **Antigen** | **n** | **Shapiro-Wilk test result (p)** | | **Levene's test result (p)** | **Student T test result (p)** | **Mann-Whitney U test result (p)** | **Welch F test result (p)** |
| --- | --- | --- | --- | --- | --- | --- | --- | --- |
|  |  |  | **SCM** | **noGF** |  |  |  |  |
| DMBC11 | Ki-67 | 3 | 0,9999 | 0,0724 | 0,3231 |  | - | 0,3027 |
| DMBC12 | Ki-67 | 4 | 0,7510 | 0,3398 | 0,5092 | 0,2883 | - | - |
| DMBC21 | Ki-67 | 4 | 0,6096 | 0,4763 | 0,7088 | 0,5440 | - | - |
| DMBC33 | Ki-67 | 2 | - | - | - | - | 1,0000 | - |
| DMBC11 | CD271 | 3 | 0,8861 | 0,7262 | 0,4495 | 0,7219 | - | - |
| DMBC12 | CD271 | 3 | 0,5491 | 0,1847 | 0,3696 | 0,8821 | - | - |
| DMBC21 | CD271 | 4 | 0,3743 | 0,4276 | 0,9273 | 0,5687 | - | - |
| DMBC33 | CD271 | 2 | - | - | - | - | 1,0000 | - |

Fig 6b:

| **Cell line** | **Treatment** | **n** | **Levene's test result (p)** | **Mann-Whitney U test result (p)** | **Welch F test result (p)** |
| --- | --- | --- | --- | --- | --- |
| DMBC12 | C | 2 | <0,0001 | 1,0000 | 0,6586 |
| DMBC12 | PLX | 2 | <0,0001 | 0,6985 | 0,4212 |
| DMBC12 | TRA | 2 | <0,0001 | 0,2453 | 0,4360 |
| DMBC21 | C | 2 | <0,0001 | 0,2453 | 0,0700 |
| DMBC21 | PLX | 2 | <0,0001 | 0,6985 | 0,4870 |
| DMBC21 | TRA | 2 | <0,0001 | 0,6985 | 0,6694 |
